# Bioactive phospho-therapy with black phosphorus for *in vivo* tumor suppression

**DOI:** 10.7150/thno.43092

**Published:** 2020-03-26

**Authors:** Shengyong Geng, Ting Pan, Wenhua Zhou, Haodong Cui, Lie Wu, Zhibin Li, Paul K. Chu, Xue-Feng Yu

**Affiliations:** 1 Materials and Interfaces Center, Shenzhen Institutes of Advanced Technology, Chinese Academy of Sciences, Shenzhen 518055, China; 2 Medical Oncology, Shenzhen People's Hospital, the Second Clinical Medical College of Jinan University, Shenzhen 518055, China; 3 Department of Physics, Department of Materials Science and Engineering, and Department of Biomedical Engineering, City University of Hong Kong, Tat Chee Avenue, Kowloon, Hong Kong, China; 4 Institute of Environment and Health, Jianghan University, Wuhan 430056, China

**Keywords:** black phosphorus, bioactive phospho-therapy, selective cell killing, two-dimensional nanomaterials, cancer therapy

## Abstract

**Background and Purpose**: Although inorganic nanomaterials have been widely used in multimodal cancer therapies, the intrinsic contributions of the materials are not well understood and sometimes underestimated. In this work, bioactive phospho-therapy with black phosphorus nanosheets (BPs) for *in vivo* tumor suppression is studied.

**Methods**: Orthotopic liver tumor and acute myeloid leukemia are chosen as the models for the solid tumor and hematological tumor, respectively. BPs are injected into mice through the tail vein and tumor growth is monitored by IVIS bioluminescence imaging. Tumor tissues and serum samples are collected to determine the suppression effect and biosafety of BPs after treatment.

**Results**: The *in vitro* studies show that BPs with high intracellular uptake produce apoptosis- and autophagy-mediated programmed cell death of human liver carcinoma cells but do not affect normal cells. BPs passively accumulate in the tumor site at a high concentration and inhibit tumor growth. The tumor weight is much less than that observed from the doxorubicin (DOX)-treated group. The average survival time is extended by at least two months and the survival rate is 100% after 120 days. Western bolt analysis confirms that BPs suppress carcinoma growth *via* the apoptosis and autophagy pathways. In addition, administration of BPs into mice suffering from leukemia results in tumor suppression and long survival.

**Conclusions**: This study reveals that BPs constitute a type of bioactive anti-cancer agents and provides insights into the application of inorganic nanomaterials to cancer therapy.

## Introduction

Cancer continues to be a major cause of mortality accounting for almost 13% of deaths worldwide 1, 2. As the most common primary tumor in the liver, hepatocellular carcinoma (HCC) is a leading cause of cancer-related death with an average 5-year survival rate of only 18% 3, 4. Currently, treatment of HCC is a big challenge due to the high rate of late diagnosis in the intermediate to advanced stages. In fact, therapeutic options such as resection and liver transplantation are only suitable for early diagnosis 5, 6. In the case of late diagnosis, local therapies such as transarterial chemoembolization have limited effects and often lead to complications 7. To make things worse, new therapies targeting the immune- checkpoint such as PD-L1 have no significant effects in clinical research of HCC 5 and the efficacy and applicability of Sorafenib (the only molecular target drug for advanced HCC worldwide) is limited 8. In this situation, systemic chemotherapy remains the mainstream treatment for HCC clinically. However, the side effects on healthy organs of traditional cytotoxic anticancer agents particularly oxaliplatin and doxorubicin (DOX) render them unsatisfactory 9 and consequently, new therapeutic agents with selective anti-tumor effects for unresectable hepatoma are highly desirable.

Nanotechnology has spurred recent development of cancer research 10, 11 and many nanomaterials have large potential in photothermal/ photodynamic therapy 12, drug loading and delivery 13, as well as multimodal theranostic treatment 14. These inorganic nanomaterials have unique physical properties and in particular, their suitable size enables passive accumulation in tumors *via* enhanced permeability and retention (EPR) 15. In fact, some nanomaterials in the unloaded state can produce certain therapeutic effects according to their intrinsic biological effects. For example, few-layer graphene has been reported to selectively kill monocytic cancer cells showing potential in chemotherapeutic treatment of leukemia 16. Intravenous administration of nanodiamonds reduces recruitment of tumor-associated macrophages and M2 macrophage polarization thus alleviating the phenotypes of tumor metastasis in mice 17. However, inorganic nanomaterials typically suffer from poor *in vivo* degradability, which can produce potential risks pertaining to long term toxicity in clinical applications.

As a new class of two-dimensional (2D) nanomaterials, black phosphorus nanosheets (BPs) have excellent physicochemical properties and large potential in biomedical applications including photothermal therapy 15, 18, photodynamic therapy 19, drug/biomolecule delivery 20, 21, bioimaging 22, and multimodal therapy of cancer 23-28. Recently, the intrinsic effects of BPs on cells and systems have been studied. For example, BPs can induce the generation of intracellular reactive oxygen species (ROS) and disrupt the cell membrane integrity 29. BPs have also been reported to induce immunotoxicity and immune perturbation in macrophages 30. Compared to other inorganic nanomaterials, the uniqueness of BPs lies in the good degradability in the presence of water and oxidative stress to produce phosphate anions that are the major degradation products 31, 32. Although phosphate anions as a physiological buffering agent have good biocompatibility, instantaneous increase of cytosolic phosphate anions affects cellular ATP hydrolysis 33, 34 and induces cell apoptosis 35, 36. We have recently observed that owing to the stronger intracellular oxidative stress and accelerated energy metabolism in cancer cells compared to normal cells 37, 38, high intracellular uptake and rapid degradation of BPs produce instantaneous increase in the cytosolic phosphate anions level, subsequently killing cancer cells 39. In many cases, *in vitro* studies produce promising results, but subsequent *in vivo* studies fail to reproduce the efficacy and/or biosafety due to multiple and complex metabolic processes taking place in the body. Therefore, considering the complex physiological environment and instability of BPs, the anti-tumor bioactivity of BPs requires a systematic study *in vivo*.

In this work, the *in vivo* tumor suppression effects of BPs on HCC are investigated in details. In addition to solid tumors, acute myeloid leukemia which is one of the most common hematological tumors is selected to evaluate the universal nature of BPs in cancer therapy. The most common chemotherapy drugs for liver cancer include gemcitabine, doxorubicin, oxaliplatin, cisplatin, 5-fluorouracil (5-FU), capecitabine, and mitoxantrone 40, 41. The drugs often used to treat acute myeloid leukemia consist of a combination of cytarabine (cytosine arabinoside or Ara-C) and an anthracycline drug such as daunorubicin, idarubicin or DOX 42. Therefore, DOX as a first-line chemotherapy drug for these two cancer types is adopted as a positive control in the study and BPs with a lateral size of about 180 nm serve the bioactive phospho-therapeutic agents. *In vitro* studies show that BPs selectively kill human hepatocellular carcinoma cells (HepG2) compared to human normal liver cells (QSG-7701). After intravenous injection into mice with the orthotopic liver tumor, a large amount of BPs accumulates passively in the tumor site resulting in effective suppression of tumor growth and longer survival time superior to DOX. In addition, BPs exhibit tumor inhibition of the acute myeloid leukemia-bearing mice. The *in vivo* results indicate the immense potential of BPs in cancer therapy.

## Materials and Methods

### Materials

The bulk BP crystals were purchased from MoPhos (China) and stored in a dark Ar glove box. N-methyl-2-pyrrolidone (NMP) (99.5%, anhydrous) was purchased from Aladdin Reagents and DOX was purchased from Melone Pharmaceutical Co., Ltd. (Dalian, China). The phosphate buffered saline (PBS, pH 7.4), trypsin-EDTA, RPMI-1640, DMEM media, fetal bovine serum, and penicillin-streptomycin were purchased from Gibco Life Technologies (AG, Switzerland). The Cell Counting Kit-8, Annexin V-FITC Apoptosis Detection Kit, Cell Cycle and Apoptosis Analysis Kit, 4% parafoemaldehyde (PFA) and PBST were bought from Beyotime (Shanghai, China) and XenoLight D-Luciferin-K^+^ Salt Bioluminescent Substrate was purchased from PerkinElmer (USA). The antibodies used in immunostaining were purchased from Abcam (Cambridge, UK) and Cell Signaling Technology (USA). All the other chemicals were analytical reagent grade and used without further purification.

### Preparation and characterization of BPs

The BPs were prepared by a modified liquid exfoliation technique established by our group. Briefly, 25 mg of the BP crystal powder were added to 25 mL of NMP and sonicated with a sonic probe in an ice bath for 10 h at 1080 W (2 s on and 5 s off), followed by bath sonication for another 10 h at 300 W. The dispersion was centrifuged at 9000 rpm for 10 min to remove oversized BP sheets and the supernatant containing BPs was centrifuged at 15000 rpm for 10 min. The precipitate was collected and re-suspended in NMP for storage. The BPs were rinsed with ethanol and water prior to experiments.

The TEM images were obtained from JEM-3200FS (JEOL, Japan) at an acceleration voltage of 200 kV. The size distribution and zeta potential of the BPs were determined by DLS using the Zetasizer 3000 HAS (Malvern Instruments Ltd., UK). XPS was carried out on the Thermo Fisher ESCALAB 250Xi XPS and Raman scattering was performed on the Jobin-Yvon LabRam HR-VIS high-resolution confocal Raman microscope (Horiba, Japan) with the 633 nm laser as the excitation source. The concentration of BPs was determined by inductively-coupled plasma atomic emission spectroscopy (IRIS Intrepid II XSP, Thermo Electron Corporation).

### Cell culture

The cell lines were purchased from China type culture collection (CTCC) through the American Type Culture Collection (ATCC). The human liver cancer cells (HepG2) were cultured in DMEM medium supplemented with 10% (v/v) fetal bovine serum and 1% (v/v) penicillin/streptomycin under humidified conditions of 5% CO_2_ at 37 °C. The normal human liver cells (QSG-7701) and human promyelocytic leukemia cells (HL-60) were cultured in RPMI-1640 media supplemented with 10 and 20% (v/v) fetal bovine serum, respectively, under the same conditions.

### Cell cytotoxicity assay

The cell cytotoxicity was determined using the CCK-8 assay. The cells were seeded on 48-well plates at a density of 5 × 10^4^ cells/mL. After incubation for 24 h, the culture medium was replaced by a medium containing serial dilutions of BPs and cultured for another 24 or 48 h. Since HL-60 was the suspension with the cultured cells, the culture medium containing BPs was stirred in order to facilitate uptake of BPs during incubation. At different time points, the cells were washed with PBS and a medium containing the 10% (v/v) CCK-8 solution was added to each well followed by incubation for 2 h at 37 ^o^C. A microplate reader (FilterMax F5, Molecular Devices, USA) was used to measure the absorbance at 450 nm. The untreated cells were the control group.

### Intracellular uptake and degradation of BPs

Intracellular trafficking of BPs was evaluated by confocal laser scanning microscopy (Leica TCS SP5-II). The FITC-labeled BPs were prepared by adding 0.1 mg/mL of FITC to the BPs solution and stirred for 4 h at room temperature in the dark. The excess dye molecules were removed by centrifugation and washed away with water more than 5 times until fluorescence signals were not detected from the supernatant. The HepG2 and QSG-7701 cells (5 × 10^4^ per well) were seeded on confocal dishes and cultured overnight. The cells were incubated with medium containing BPs (4 μg/mL) for different time intervals (6, 12, and 24 h), rinsed with PBS, stained with lysosome dyes (LysoTracker Red DND-99, 50 nM) at 37 °C, and incubated for 1 h. Afterwards, the cells were rinsed with PBS and stained with Hoechst 33342 for 10 min and the live cells were imaged under a fluorescence microscope. Degradation of BPs by HepG2 and QSG-7701 cells were monitored by Raman scattering microscopy. The cells were cultured on a climbing glass on 24-well plates for 24 h (5 × 10^4^ cells per well). The culture medium was replaced by a medium containing BPs (4 µg/mL) and cultured for 6, 12, or 24 h. The cells were fixed with 4% PFA and transferred to a slide glass, followed by Raman mapping using the BPs characteristic A2 g Raman peak at 462 cm^-1^.

The mechanism of cellular uptake was investigated by pre-treatment at a low temperature (4 ^o^C), amiloride (2 mM), MβCD (10 mM), and sucrose (450 mM) for 0.5 h and incubation with FITC-labeled BPs (4 µg/mL) for 2 h. The cellular uptake of BPs was examined by confocal laser scanning microscopy (LEICA-TCS SP5II, Germany). To quantify the intracellular fluorescence intensity, the cells were collected and lysed with the RIPA lysis buffer for 0.5 h on ice before measuring the fluorescence intensity (Hitachi F-4600, Japan).

### Cell apoptosis assay

The cells were grown on 12-well plates at a density of 1 × 10^5^ cells/mL and treated with different concentrations of BPs (0 to 32 µg/mL). After 24 or 48 h, the cells were harvested by 0.25% trypsinization, centrifuged at 1000 rpm for 5 min, and washed with ice cold PBS. Apoptosis was assessed on an Annexin V-FITC Apoptosis Detection Kit according to the manufacturer's instructions (Beyotime, China). The stained cells were analyzed by flow cytometry (FACSCalibur, BD, USA) and data analysis was performed using the FlowJo software. A quadrant analysis was performed and cells stained positive for Annexin V-FITC and/or PI were designated as apoptotic and unstained cells were designated as alive.

### Western blot analysis

The cells were cultured on 6-well plates to approximately 80% confluence. The culture medium was replaced with fresh medium containing BPs (4 μg/mL) and incubated for 24 or 48 h. After washing twice with PBS, the cells were harvested by 150 μL of the M-PER mammalian protein extraction reagent (Thermo Fisher Scientific). The cell lysates were centrifuged at 12000 rpm for 10 min at 4 °C and the supernatants were collected. The protein concentrations in the supernatants were quantified by the Bradford assays (Bio-Rad). The denatured proteins samples were analyzed by 12% SDS-PAGE at 80 V for 90 min and blotted to PVDF membranes (Millipore) at 175 mA for 90 min in an ice bath. The blots were blocked for 1 h using 5% nonfat milk in the PBST (PBS containing 0.1% Tween 20) buffer at room temperature and incubated overnight at 4 ^o^C with the primary antibodies: Caspase-3/active caspase-3 (CST, 9665S), p53 (Abcam, ab32389), p62 (Abcam, ab109012), LC3 (Abcam, ab51520), or beta Actin (Abcam, ab8229). After extensive washing with PBST buffer, the blots were incubated with a goat anti-rabbit horseradish peroxidase (HRP)-conjugated secondary antibody (CST, 7074S) for 1 h at room temperature. The blots were then incubated with ECL reagent (Thermo Fisher Scientific) and the densities of all bands were quantified using the Chemiluminescent Imaging System (6100c, Tanon, China). The expression of beta Actin was used as the protein loading control.

In the *in vivo* experiments, the orthotopic liver tumors excised from the mice after treatment were minced and homogenized in the protein lysate buffer. The debris was removed by centrifugation and the levels of caspase-3, p62 and LC3 in lysates were measured as aforementioned.

### Tumor models and treatment

The Balb/c nude mice (female, 18-20 g, 4-6 weeks old) and NOD/SCID mice (female, 20-25 g, 4-6 weeks old) were purchased from Charles River Laboratory Animal Technology Co., Ltd. (Beijing, China) and kept in specific pathogen-free conditions in laminar flow cabinets (18-22 °C, 50-70% relative humidity, 12 h light-dark cycle). The animals had free access to tap water and standard pellet food. All the animal experiments were conducted in accordance with the Institute's Guide for the Care and Use of Laboratory Animals and animal experiment protocols were approved by the Administrative Committee on Animal Research of the Shenzhen Institutes of Advanced Technology, Chinese Academy of Sciences.

The orthotopic liver cancer model was established as follows. The anesthetized Balb/c nude mice were placed in supine position and a midline incision was made to expose the liver. For tumor implantation, the lower surface of the left liver lobe was chosen because this lobe could be easily exteriorized *in vivo* and allowed intravital microscopic observation due to the planar surface. 15 µL of PBS containing 2 × 10^6^ luciferase-transfected HepG2 cells was directly implanted into the parenchyma of the left liver lobe using a micro-injector with 32-gauge needle (Hamilton, Switzerland). The puncture site was occluded by acrylic glue to completely abolish peritoneal tumor spread, and then the liver and abdomen was closed with silk sutures. The acute myeloid leukemia tumor model was established by injecting 1 × 10^7^ luciferase-transfected HL-60 cells (dispersed in 100 µL of PBS) into NOD/SCID mice from the tail vein. The maximum tolerated dose (MTD) was determined according to that causing less than 15% body weight loss. The MTD of DOX in the Balb/c mice was 5-10 mg/kg 43-45. In our study, the healthy Balb/c nude mice and NOD/SCID mice were intravenously administrated by a single injection with 5 or 8 mg/kg (3 mice in each group) to evaluate the MTD of DOX. As for the MTD of BPs, the injection doses were 2.5 or 5 mg/kg and the body weight changes of the mice were recorded.

### Biodistribution analysis

To visualize the BPs biodistribution *in vivo*, fluorescent labeled BPs were prepared by adding 0.1 mg/mL of Cy5.5 to the BPs solution and stirred for 4 h at room temperature in the dark. The excess dye molecules were removed by centrifugation and washed away with water more than 5 times until the fluorescence signal of supernatant could no longer be detected. The Cy5.5-labeled BPs were re-suspended in PBS. Following a single intravenous injection of Cy5.5-labeled BPs (2.5 mg/kg BPs for each orthotopic liver tumor-bearing mouse), the mice were imaged at 1, 3, 6, 12, 24 and 48 h post-injection using an IVIS Spectrum (Caliper IVIS Spectrum, PerkinElmer, USA). In the *ex vivo* fluorescence imaging experiments, the BPs-treated mice were killed by cervical dislocation and the heart, liver, spleen, lung, and kidney were collected and imaged immediately afterwards. The fluorescence intensity was simultaneously quantified using Living Image 4.2.

In a separate experiment, the BPs were directly examined in the tumor site by Raman scattering mapping. The liver was removed and tumor tissue was subdivided at different time points post intravenous injection of 100 µg BPs per mouse. 0.2 g of the tumor pieces were cut into smaller pieces with a pair of scissors. The Cell and Tissue Lysis Buffer (Beyotime, China) was added and homogenized until fully lysis. The solution was centrifuged at 3000 rpm for 5 min to remove oversized tissues and the supernatant was centrifuged at 15000 rpm for 10 min. The precipitate was transferred to a slide glass and Raman intensity mapping was performed based on the BPs characteristic A2 g Raman peak at 462 cm^-1^.

### Pharmacokinetic analysis

Free DOX and DOX-labeled BPs at doses of 5 mg DOX/kg body weight were administered into Balb/c nude mice *via* the tail vein. At various time points (0.25, 0.5, 1, 2, 4, 8, 12, 24, and 48 h) after injection, 10 μL of blood were collected from the tail vein and the concentration of DOX in the plasma was determined by high-performance liquid chromatography (HPLC) as previously reported 46.

### *In vivo* antitumor study

To assess the antitumor effects, the orthotopic liver tumor-bearing Balb/c nude mice were randomly divided into three groups each containing of 10 mice. One group (control) was intravenously injected with PBS and the other groups (treatment) were subjected to antitumor treatment *via* intravenous injection of DOX (5 mg/kg) or BPs (2.5 mg/kg). The mice were treated with seven doses on day 0, 7, 14, 21, 28, 35, and 42. Tumor development was monitored by acquiring the bioluminescent images of the abdomen of the orthotopic liver cancer mice and calculated by the bioluminescent intensity. In *in vivo* monitoring of light emission, the mice were intraperitoneally injected with 150 mg/kg D-luciferin (200 μL of a 15 mg/mL solution dissolved in PBS) and after 5 min, the mice were imaged using an IVIS Spectrum (Caliper IVIS Spectrum, PerkinElmer, USA) for an exposure time of 30 s and the bioluminescent signals were simultaneously quantified with the Living Image 4.2. At the end of the treatment, four mice were randomly selected from each group and sacrificed on day 49 to measure the tumor weight and tumor spread to the lung. The weight of the tumor tissue was calculated by subtracting the weight of the liver from the non-tumor-bearing mice. The body weight of mice was measured regularly and the survival data were displayed as Kaplan-Meier plots.

As for the therapy of acute myeloid leukemia, the mice (n = 5) were injected intravenously with BPs on day 0 and 3 with doses of 2.5 mg/kg and the control group was injected with the same volume of PBS. IVIS Spectrum was used to monitor the changes of tumor. The body weight and the survival time were recorded.

### Biosafety evaluation

After intraperitoneal injection of 40 mg/kg pentobarbital sodium, the blood samples were collected by intracardiac puncture to measure the serum levels of the liver enzymes (ALT, AST and ALP) and renal functions (CRE, UREA and UA) with the activity Assay Kit based on an automatic biochemical analyzer (Mindray BS-180VET, China). The main organs including the heart, liver, spleen, lung, and kidney were isolated at day 49 and the organs were fixed with 4% poly-formaldehyde (PFA) for H&E staining and TUNEL assay.

### Histopathological and TUNEL analysis

The heart, liver, spleen, lung, kidney, and tumor of the orthotopic tumor-bearing mice were paraffin-embedded, sectioned at 5 μm thick, and stained with H&E. The sections were examined by optical microscopy (Olympus IX71, Japan).

TUNEL staining was performed using the *in situ* death-detection POD kit (Roche Diagnostics) on paraffin-embedded orthotopic tumor sections according to the manufacturer's instructions. DAPI counterstaining was used to quantify cells with intact nuclei and the apoptotic HepG2 cells labeled with fluorescein dye were examined by fluorescence microscopy (Leica DFC450C).

### Statistical Analysis

All the results were presented as mean ± SD. The differences among groups were determined using a two way (time*treatment) repeated measures ANOVA analysis followed by Tukey's post-test. All the statistical analyses were performed with the IBM SPSS 19 software. *p < 0.05, **p < 0.01, and ***p < 0.001.

## Results and Discussion

### Characterization of BPs

**Figure 1:**
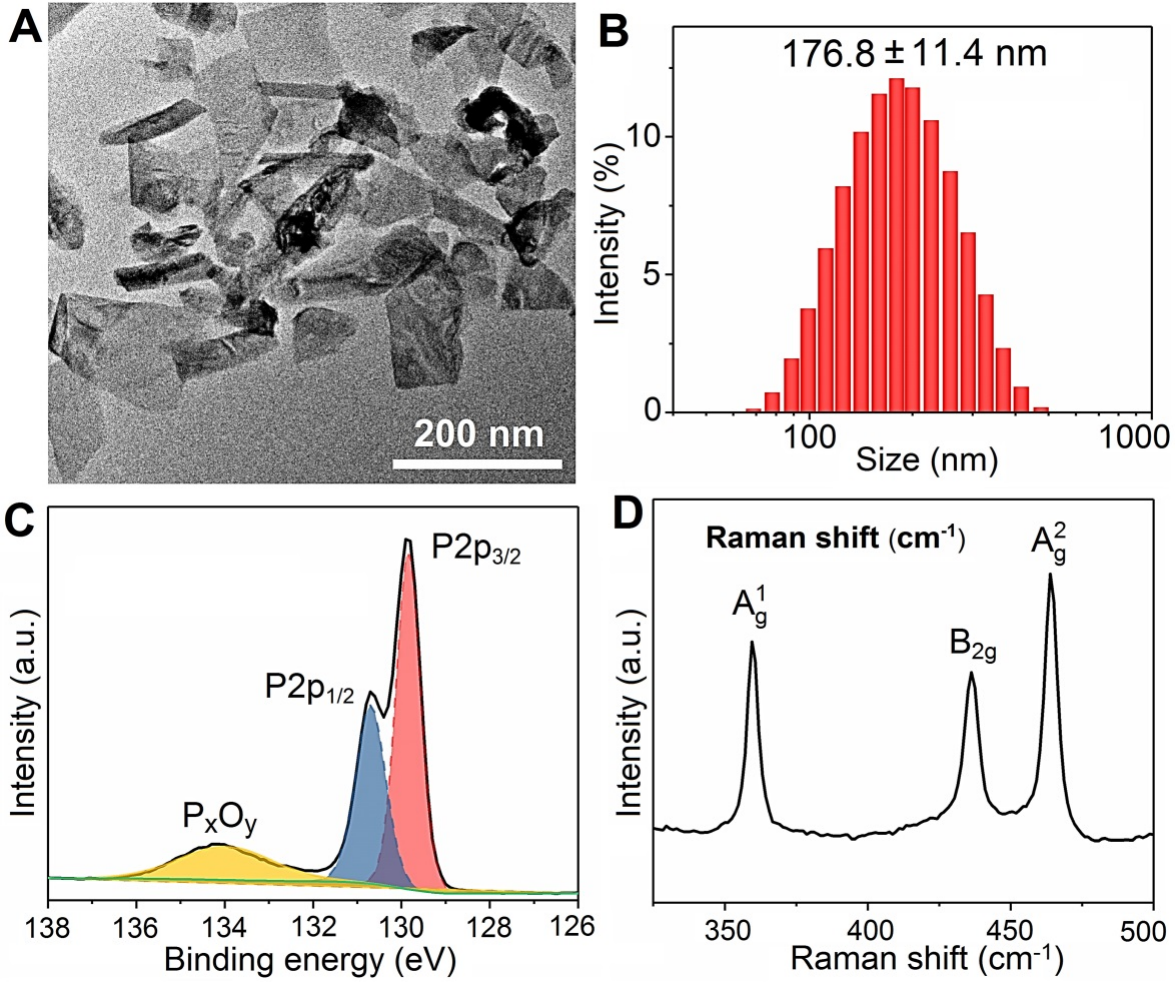
** Characterization of BPs. (A)** TEM image; **(B)** Size distribution measured by DLS; **(C)** P 2p XPS spectrum; **(D)** Raman scattering spectrum.

The BPs are prepared by liquid exfoliation 47, and the transmission electron microscopy (TEM) image reveals good dispersibility and a sheet-like morphology with a lateral size of 100-200 nm (Figure 1A). According to dynamic light scattering (DLS), the mean hydrodynamic radius of BPs is 176.8 ± 11.4 nm (Figure 1B) and the zeta potential is -26.8 ± 1.4 mV (Figure S1). As shown in Figure 1C, the doublet peaks at 129.9 and 130.8 eV in the XPS spectrum correspond to P 2p_3/2_ and P 2p_1/2_, respectively, in agreement with crystalline BP 48-50. The Raman scattering spectrum in Figure 1D shows the three characteristic Raman peaks of BP in 359.5, 436.3 and 463.9 cm^-1^ confirming successful preparation of BPs.

### *In vitro* cytotoxicity assay of BPs

**Figure 2:**
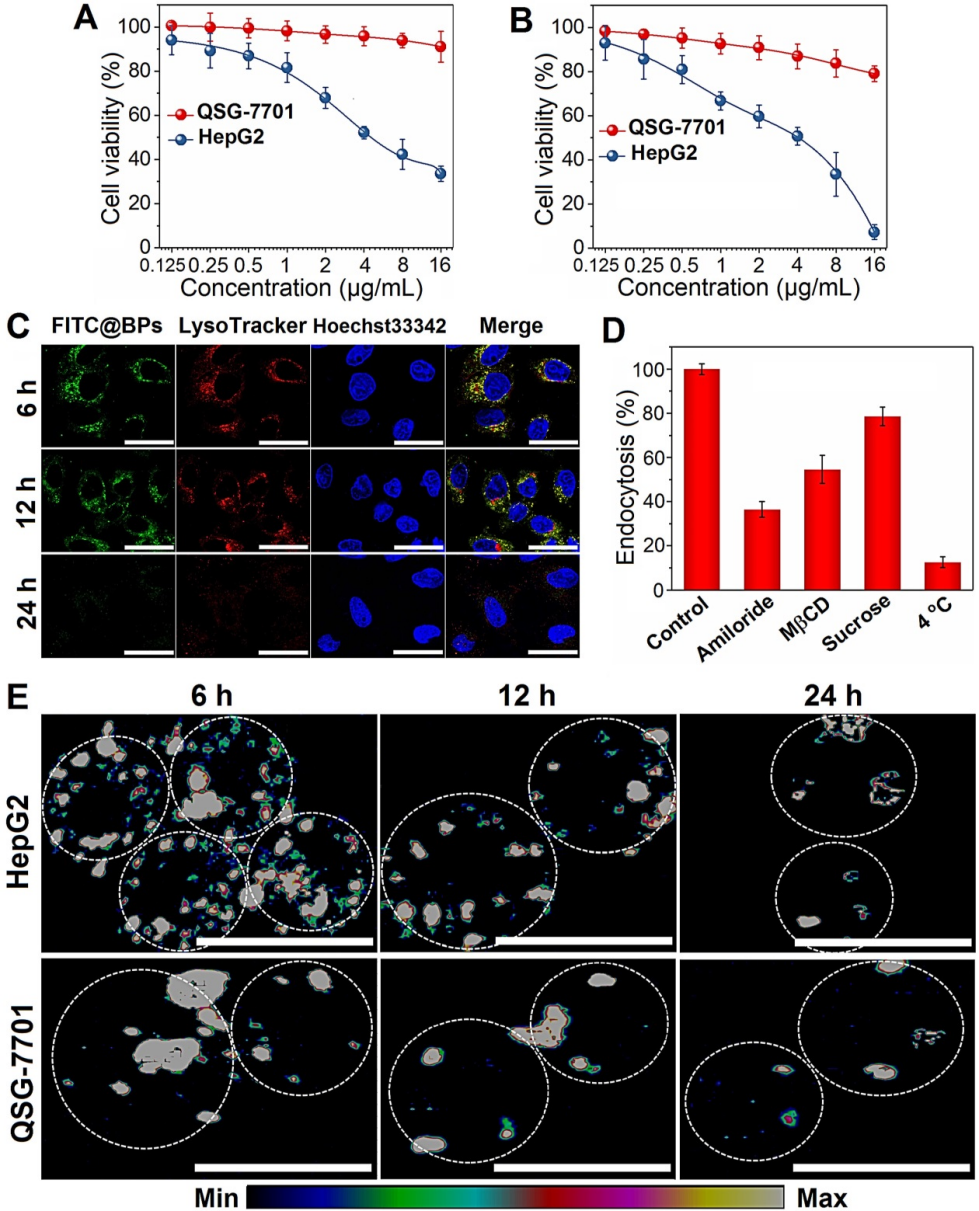
** Cellular uptake and degradation of BPs. (A and B)** Relative cell viability of HepG2 and QSG-7701 cells as a function of BPs concentration after treatment for 24 or 48 h, respectively (n = 6); **(C)** Confocal fluorescence images of HepG2 cells treated with BPs (4 µg/mL) for different time intervals. The green and red fluorescence images show FITC and LysoTracker Red DND-99, respectively (scale bar = 20 µm); **(D)** Endocytosis inhibition of BPs by HepG2 cells after treatment with different inhibitors and low temperature of 4 ^o^C; **(E)** Intracellular degradation of BPs (4 µg/mL) by HepG2 and QSG-7701 cells monitored by Raman mapping of the characteristic A2 g peak of BPs (scale bar = 20 µm).

The stability of BPs in the cell culture medium (RPMI-1640 and DMEM) containing 10% serum is evaluated. No aggregation or precipitation is observed within 48 h but BPs undergo degradation in the medium (Figure S2). The effects of BPs on the viability of cancerous HepG2 and normal QSG-7701 cells are evaluated by the Cell Counting Kit-8 (CCK-8). BPs inhibit cell viability in a dose- and time-dependent manner. After incubation with 16 µg/mL of BPs, the survival rates of HepG2 cells decrease to 34% and 6% after 24 and 48 h, respectively, but BPs do not affect the viability of QSG-7701 cells (Figure 2A, B). To clarify selective killing of cancer cells by BPs, the uptake and intracellular trafficking of BPs by HepG2 and QSG-7701 cells are determined by laser scanning confocal microscope. The nano-system would be transported to lysosomes after being internalized into the cells. The intracellular trafficking of BPs followed by endocytosis is detected by staining the BPs treated cells with LysoTracker dyes. After incubation for 6 h, strong green fluorescence signals are observed from the FITC-labeled BPs in the HepG2 cells and the signals merge well with lysosomes which are marked by the LysoTracker (Figure 2C). In comparison, the signals from BPs decrease sharply from 12 h and disappear from 24 h, suggesting accelerated degradation of BPs under the acidic conditions in lysosomes. Interestingly, the fluorescence signals in lysosome also disappear after 24 h (Figure 2C). This is because degradation of BPs releases a large amounts of phosphate ions which elevate the osmotic pressure and induce endosome bursts 51. In contrast, there are little fluorescence signals from FITC in the QSG-7701 cells during the 24 h-incubation period (Figure S3) indicating low intracellular uptake of BPs due to the slow energy metabolism in normal QSG-7701 cells. Furthermore, Raman mapping is carried out to directly measure the intracellular signals of BPs (Figure 2E) and higher uptake and more rapid degradation of BPs are also observed in HepG2 cells to cause selective killing of HepG2 cells.

Internalization of BPs-based delivery platforms has been reported mainly through endocytic pathways 21, 52 and the mechanism of cellular uptake of BPs by HepG2 cells is investigated here using different endocytosis inhibitors: amiloride, methyl-β-cyclodextrin (MβCD), and sucrose to verify the micropinocytosis, caveolae-mediated endocytosis, and clathrin-mediated endocytosis, respectively (Figure 2D and Figure S4). Incubation at 4 °C inhibits 87.5% uptake of BPs suggesting that internalization of BPs is mainly through the energy-dependent endocytic process. The endocytosis efficiency decreases significantly in the cells pre-treated with amiloride and MβCD, while slight endocytosis inhibition is observed after the sucrose treatment. The results suggest that micropinocytosis and caveolae-dependent endocytosis are the main pathways for the endocytic uptake of BPs by HepG2 cells.

### Cell killing mechanism of BPs

**Figure 3:**
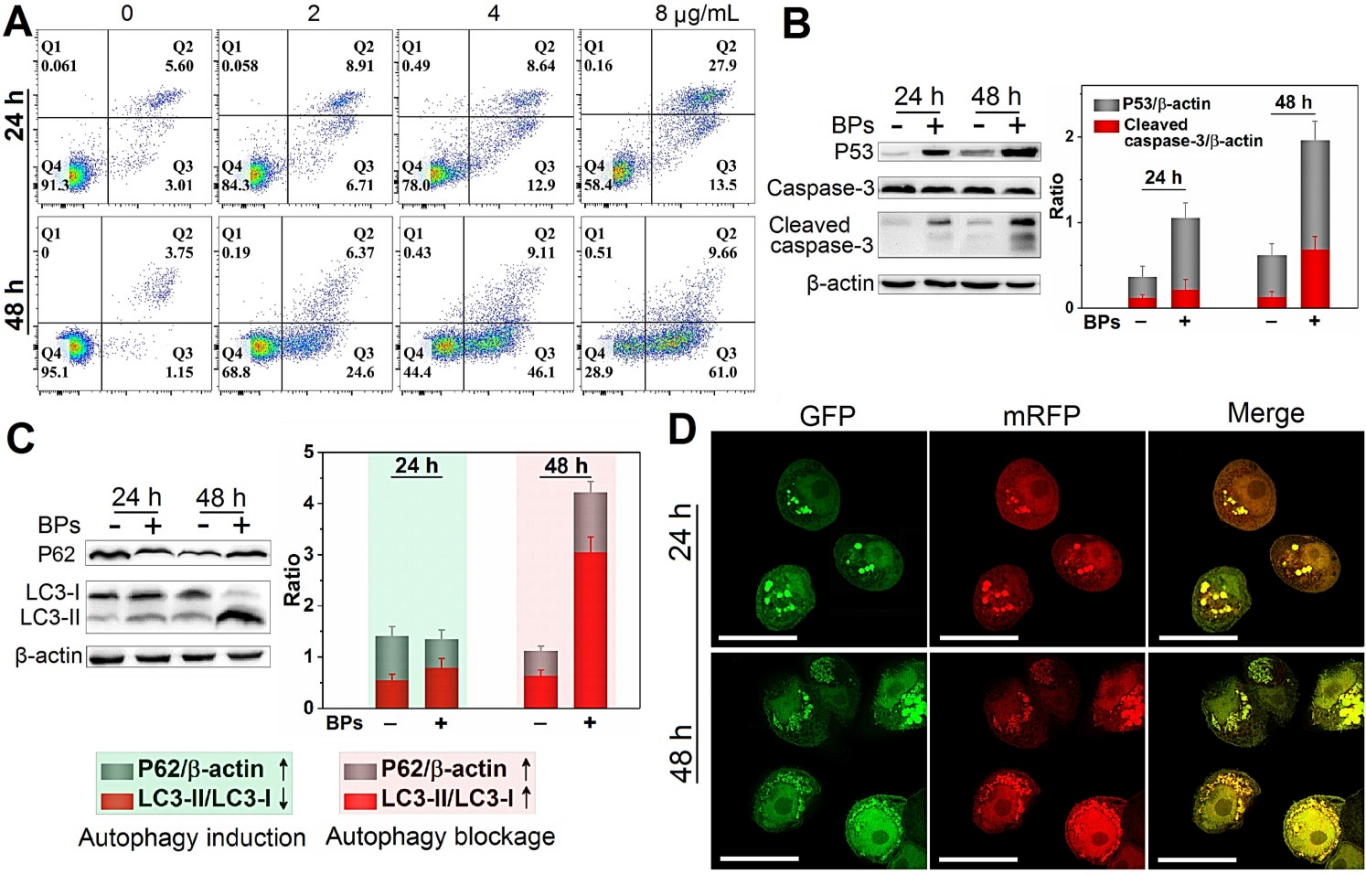
** Cell killing mechanism of BPs. (A)** Apoptosis assay of HepG2 cells by Annexin V/PI staining after treatment with different concentrations of BPs for 24 or 48 h; **(B)** Immunoblotting and semi-quantified analysis by Image J for apoptosis-related proteins (caspase-3 and p53); and **(C)** Autophagy-related proteins (LC3-I, LC3-II and p62) extracted from the HepG2 cells after treatment with 4 µg/mL BPs for 24 or 48 h (n = 3); **(D)** Confocal fluorescent images of mRFP-GFP-LC3-transfected HepG2 cells after treatment with BPs (4 µg/mL) for 24 or 48 h with mRFP used to label autolysosomes (red spots) and the yellow spots (combination of mRFP and GFP fluorescence signals) indicating the autophagosomes. The scale bar is 20 µm.

The death mechanisms of HepG2 cells by BPs are investigated. Flow cytometry analysis by Annexin V/PI staining reveals that apoptosis (Q2 + Q3) occurs in most of the HepG2 cells after treatment with BPs (8 µg/mL) for 48 h (Figure 3A) but the percentage of apoptotic QSG-7701 cells is only 16% under the same conditions (Figure S5). Western blot analysis shows that cleaved caspase-3, a key apoptotic protein, appears after incubation with BPs for 24 h and the corresponding protein expression level increases significantly after 48 h (Figure 3B). Compared to the control group, the expression of pro-apoptotic marker protein, p53, is also strongly up-regulated by BPs (Figure 3B). The elevated expression of the two marker proteins indicates that the chemotherapeutic efficiency of BPs stems from activation of the apoptosis signal pathway (the first identified programmed cell death). Besides apoptosis, autophagy is known as a type II programmed cell death pathway and generally activated in response to the chemotherapeutic treatment of solid tumors 53, 54. In our study, up-regulation of autophagy-related protein LC3-II and down-regulation of p62 are observed after 24 h (Figure 3C), suggesting autophagy induction by BPs. As incubation continues, a large quantity of LC3II is expressed but the increased expression of p62 suggests that degradation of p62 is blocked after 48 h. To further monitor the autophagic flux inhibition, a tandem fluorescent-tagged LC3 reporter (mRFP-GFP-LC3) is employed. BPs-induced mRFP-GFP-LC3-positive autophagosomes (yellow spots) are observed after 24 h and show the typical characteristics of increased autophagosome-lysosome flow (Figure 3D). However, there are a large number of yellow spots in the cytoplasm after 48 h instead of gradual degradation as in the normal autophagy process. In comparison, few yellow spots are observed from the control group (without treatment of BPs) during 48 h (Figure S6) confirming that BPs induce autophagy in HepG2 cells and block the autophagic flux after long-term incubation. In fact, the impaired autophagic flux has been regarded as a promising mechanism of various chemotherapeutic agents in cancer therapy 55 such as chloroquine and CA-5f 56-59. All in all, our results demonstrate that BPs are efficiently internalized by HepG2 cells and induces cell death through apoptosis and autophagy combined pathways.

### *In vivo* tumor targeting and biodistribution of BPs

To study the *in vivo* therapeutic potential of BPs, the biodistribution is assessed using an orthotopic liver tumor model established by directly injecting luciferase-transfected HepG2 cells into the parenchyma of the left liver lobe 60. To visualize the biodistribution of BPs *in vivo*, a near-infrared fluorescent Cy5.5 is used for BPs labeling. The loading of Cy5.5 on the BPs surface is measured by monitoring the fluorescence signal from BPs. The excess dye molecules are removed by centrifugation and washed away with water more than 5 times until the fluorescence signal cannot be detected from the supernatant (Figure S7A). After re-suspension, the sample yields a strong fluorescence signal of Cy5.5 and the fluorescence peak red-shifts from 694 to 700 nm due to formation of Cy5.5@BPs aggregates, while no fluorescence can be observed from the BPs without Cy5.5 labeling (Figure S7B). When the tumor grows to an appropriate size after two weeks, a single dose of Cy5.5-labeled BPs (20 µg BPs dispersed in 100 µL PBS) is intravenously injected into each mouse through the tail vein and IVIS fluorescence imaging is carried out to observe the distribution of BPs at different time intervals.

**Figure 4:**
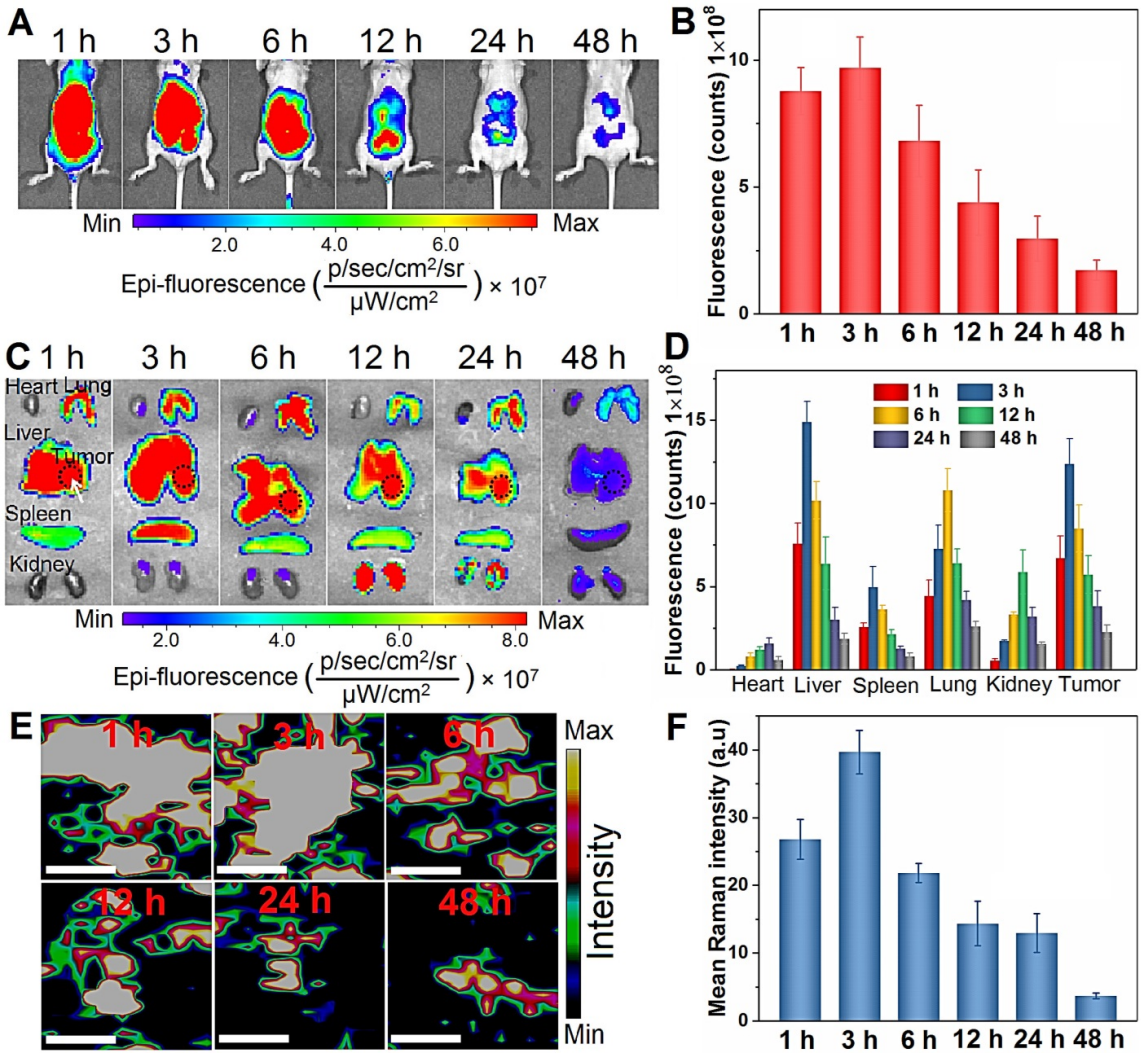
***In vivo* biodistribution analysis of BPs. (A)** *In vivo* fluorescence images of the orthotopic liver tumor-bearing nude mice treated with Cy5.5-labeled BPs at different time intervals; **(B)** Quantification of the fluorescence intensity in the liver site by ROI calculated by Living Image 4.2; **(C)** *Ex vivo* fluorescence images of the major organs from the BPs-treated mice; **(D)** Fluorescence intensity of the major organs and tumor tissue at different time points calculated by ROI; **(E)** Raman scattering maps of the characteristic A2 g peak in the tumor tissue with BPs; **(F)** Average Raman intensity of BPs calculated by the total area under the three characteristic peaks of A1 g, B_2g_, and A2 g with the error bars showing mean ± s.d. (n = 3 at each time point). The scale bar is 20 µm.

As shown in Figure 4A, strong fluorescence signals are observed in the first 6 h post-injection. Afterwards, the fluorescence intensity decreases gradually due to degradation of BPs and only weak signals are detected after 48 h. Semiquantitative analysis by ROI (region of interest) of the liver site reveals similar variations in the fluorescence intensity and maximum at 3 h post-administration (Figure 4B). To further observe the biodistribution of BPs, the mice are sacrificed to collect major organs for *ex vivo* fluorescence imaging. As shown in Figure 4C, the fluorescence signals are predominantly observed from the lung, spleen and kidney, especially in the tumor-bearing liver, and strong fluorescence maintains for 24 h. This is probably attributed to reticuloendothelial system (RES) absorption of the liver and the EPR effect on the tumor tissue. In accordance with the *in vivo* results, most of the accumulated BPs are excreted from all the organs after 48 h. ROI analysis reveals that the maximum fluorescence intensity from the tumor site is 10.3, 2.5, 1.1 and 2.1 times higher than that from the heart, spleen, lung, and kidney, respectively (Figure 4D). In fact, the main target organs for nanomaterials after intravenous administration are generally the lung, liver, and spleen 61-63. To further verify the distribution of BPs in the tumor site, the liver tumor tissues are analyzed by Raman scattering mapping after complete homogenization and lysis. Strong BPs Raman signals are observed from the liver tumor tissues in the BPs-administrated mice, suggesting high accumulation of BPs in the tumor site. The Raman signals diminish gradually and disappear after 12 h due to rapid biodegradation of BPs (Figure 4E, F). Hence, although BPs have high affinity for liver, lung and spleen, they can also accumulate passively in the orthotopic liver tumor site, which is promising for subsequent *in vivo* therapy.

The *in vivo* pharmacokinetics of BPs is examined. Since significant amounts of P exist in the animal, it is very difficult to obtain direct pharmacokinetics information of the BP-based materials. Therefore, in order to study the *in vivo* degradation and metabolism behavior of BPs, DOX is utilized to label BPs. The plasma concentration-time profiles of free DOX and DOX-labeled BPs after a single intravenous administration are determined by high performance liquid chromatography (HPLC). As shown in Figure S8, free DOX is rapidly cleared from blood circulation within 2 h, while DOX-labeled BPs exhibit much longer circulation time and much higher plasma concentration of DOX. Blood circulation of BPs obeys the typical two compartment model. The half-life of the first phase (distribution phase, with a rapid decline) is only 1.16 ± 0.18 h and then BPs circulation in blood exhibits a long second phase (elimination phase) with a half-life of 18.45 ± 1.98 h. The other pharmacokinetic parameters are also calculated by the software PKSolver using a non-compartmental model (Table S1).

### *In vivo* therapeutic efficacy of BPs on orthotopic liver tumor

**Figure 5:**
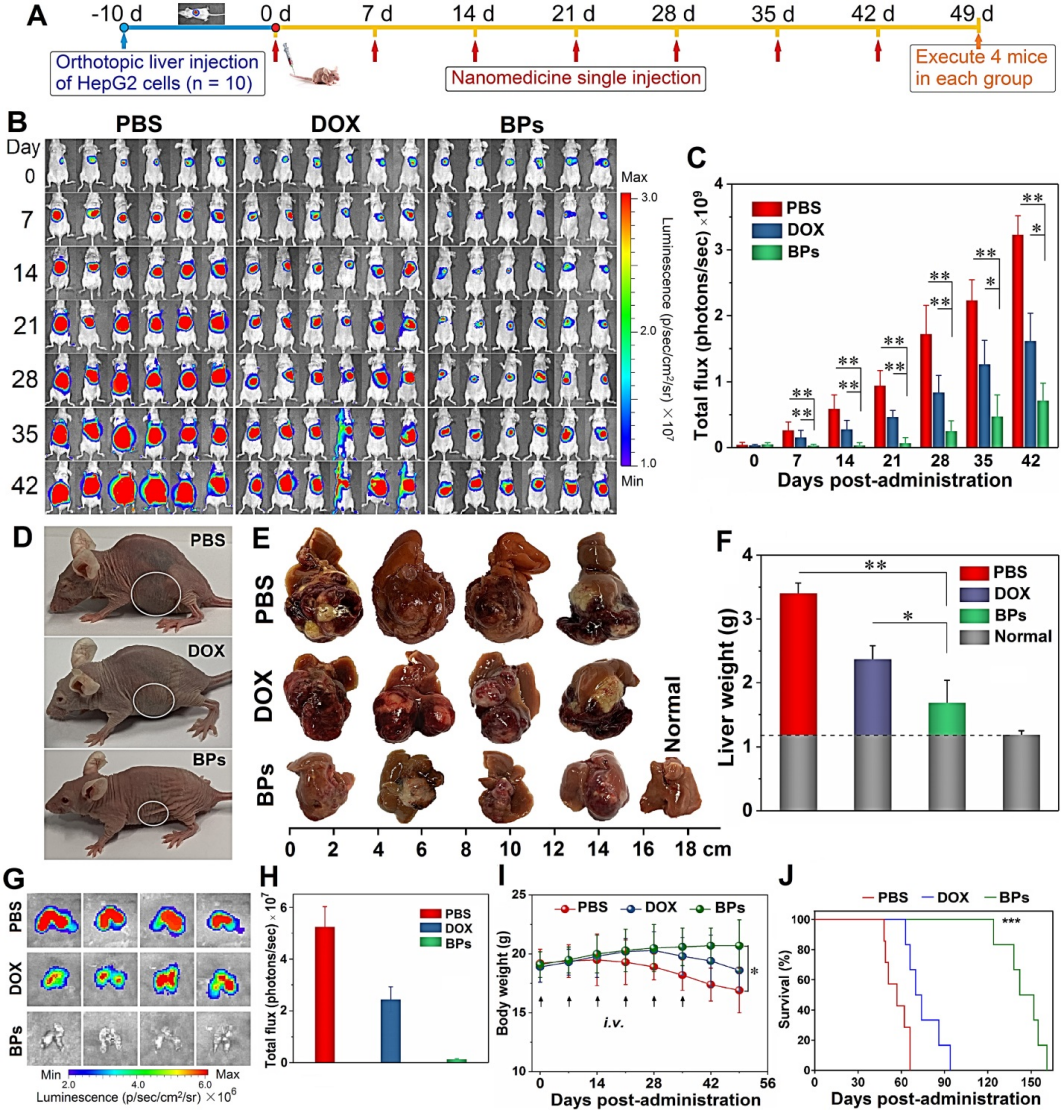
***In vivo* therapeutic effects of BPs on nude mice with the orthotopic liver tumor. (A)** Experimental schedule; **(B)** *In vivo* bioluminescence images of the orthotopic liver tumors in the different mice groups at different time points after tail vein injection of PBS (control), DOX, and BPs (n = 6); **(C)** Quantified bioluminescent intensity of the liver site measured by ROI (region of interest) using the Living Image software (n = 10); **(D)** Photographs of mice in different groups at the end of the treatment (day 49) with the tumor sites marked by ellipses; **(E)** Photographs of tumor-bearing livers of the treated mice in different groups at day 49 (n = 4); **(F)** Weight of the normal (n = 10) and tumor-bearing livers at day 49 post-different treatments (n = 4); **(G)** *Ex vivo* bioluminescence images; **(H)** Quantification of the bioluminescence intensity of lung metastases after different treatments after 49 days (n = 4); **(I)** Body weight of the mice during the 49-day period with different treatments (n = 10); **(J)** Survival curves of the mice in different groups (n = 6). *p < 0.05, **p < 0.01, ***p < 0.001 by a two way (time*treatment) repeated measures ANOVA using SPSS, significantly different from PBS or DOX.

Having demonstrated selective killing of HepG2 cells *in vitro* and passive tumor-targeting ability of BPs *in vivo*, the anti-tumor activity is further evaluated. The orthotopic tumor model is implemented to simulate the actual conditions of clinical cancer therapy and to provide a model to assess the metastatic effects in comparison with subcutaneous xenograft tumors and Figure 5A illustrates the procedures of the *in vivo* experiments. The study is initiated 10 days after implantation of luciferase- transfected HepG2 cells into the liver lobe. The maximum tolerated dose (MTD), which is determined by that causing less than 15% body weight loss of the administrated dose is calculated to be 5 mg/kg and 2.5 mg/kg for DOX and BPs, respectively (Figure S9). The tumor-bearing mice with similar bioluminescent intensity are divided randomly into three groups (10 mice per group) and intravenously injected with 100 μL of PBS, DOX (5 mg/kg), and BPs (2.5 mg/kg), respectively, once a week for 6 consecutive weeks.

Since the volume of the orthotopic liver tumor cannot be measured directly, tumor progression is noninvasively monitored by measuring the bioluminescent intensity at the tumor site. After 42 days since first administration, very strong bioluminescent signals with large areas are observed from the PBS-treated group, indicating that tumors not only develop in the liver but spread to the abdomen (Figure 5B). Compared to the PBS-treated group, although the treatment with DOX partially suppresses the enhancement of the bioluminescent signals, the signals are still throughout the liver site and obvious tumor metastasis is observed in two of the mice on day 42. In contrast, the bioluminescent signals of the BPs-treated group decrease in the first two weeks and then increase very slowly. According to the calculated bioluminescent signal intensity of the mice abdomen in Figure 5C, BPs treatment dramatically reduces the bioluminescent intensity in comparison with the other two groups throughout the treatment process (*p* < 0.05). At the end of the treatment, the bioluminescent intensity of the BPs-treated mice is only 22.2% and 44.2% of that of the PBS- and DOX-treated groups, respectively, suggesting that BPs have excellent therapeutic efficacy in liver tumor suppression.

Photographs of the mice after treatment show that the liver occupies nearly all the abdomen in PBS-treated group (Figure 5D). In order to measure the liver tumor weight at the end of the treatment, four mice are selected randomly from each group and sacrificed on day 49. The photographs of the excised livers in the PBS-treated group show that the tumors are so large that they protrude from the surface and occupy most of the liver (Figure 5E). The tumor volume of the DOX-treated group is slightly less than that of the PBS-treated group showing limited effectiveness of the DOX chemotherapy. In contrast, the tumor volume of the BPs-treated group decreases significantly and the liver retains the normal morphology (Figure 5E). The tumor inhibiting effect is further confirmed by comparing the tumor weight. Because the tumor grows in the middle as well as on the surface of the liver, complete removal of the tumor from the liver tissue is impracticable. Therefore, the tumor weight is calculated by subtracting the weight of the normal liver from the weight of the tumor-bearing liver. As shown in Figure 5F, administration of BPs reduces the tumor weight from 2.2 g (PBS-treated group) to 0.5 g and the result is superior to that observed from the DOX-treated group (*p* < 0.05) at the end of the treatment.

Liver tumor may undergo metastasis and lung is the most common site for extrahepatic spreading 64. To examine lung metastasis, the mice are intraperitoneally injected with D-luciferin and sacrificed after 49 days before the lung is harvested for *ex vivo* IVIS imaging. The bioluminescent images in Figure 5G show obvious lung metastasis in the PBS- and DOX-treated groups, even though DOX shows moderate suppression in the bioluminescent intensity (Figure 5H). In contrast, administration of BPs results in total inhibition of lung metastases at the end of the treatment demonstrating significant inhibition of orthotopic liver tumor growth and consequent elimination of lung metastases in response to BPs.

The health state and survival rate of the remaining six mice in each group are also monitored. Morphological observation shows that the mice in the PBS- and DOX-treated groups are listless at the end of the treatment (Figure 5D). In contrast, morphology of the BPs-treated mice is similar to the normal ones. Body weight decrease is observed in the PBS- and DOX-treated groups beginning from 14 or 28 days post-administration, respectively, while no significantly effect on the body weight is observed from the BPs-treated group (Figure 5I). There is a statistical difference in the body weight between the BPs- and PBS-treated groups after the treatment (*p* < 0.05). The BPs-treated mice survive for over 120 days without a single death, whereas all the mice in the PBS- and DOX-treated groups die within 66 and 94 days, respectively (Figure 5J). The average survival time is extended by at least two months by treatment with BPs and 50% of the mice in the BPs-treated group survive for over 150 days. The reason why the anti- tumor efficacy is superior to DOX may be that long blood circulation of BPs delays macrophage clearance in reticuloendothelial systems (RES) favoring enhanced tumor targeting by the EPR effect (Figure S8 and Table S1). However, the *in vivo* efficacy of BPs in liver cancer needs improvement by other therapeutic agents. For example, combination therapy with nanodiamonds and arsenic trioxide results in 100% survival rate in 5 months post-therapy with reduced advanced liver carcinoma-associated symptoms 65.

### Molecular mechanism of BPs therapy

**Figure 6:**
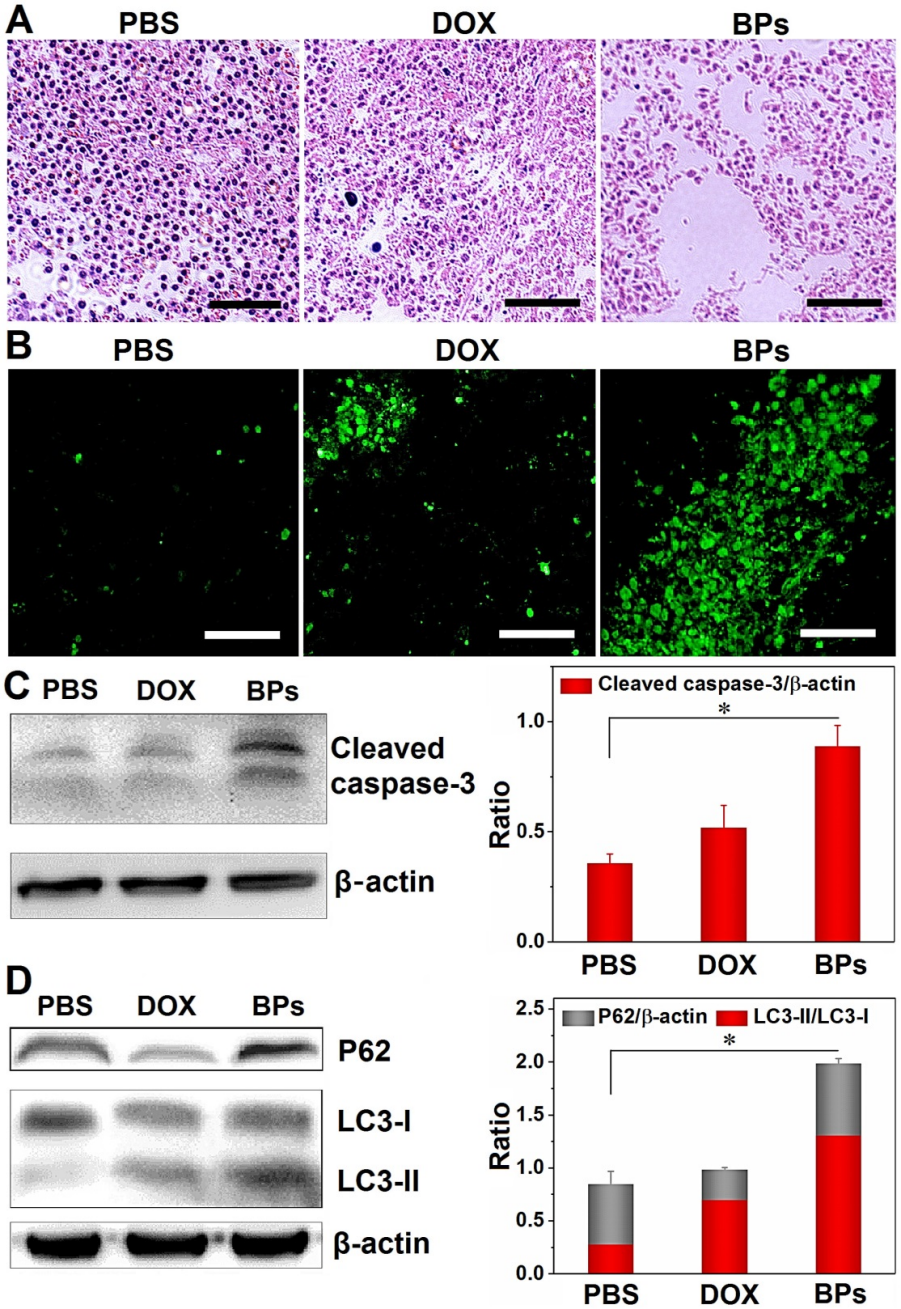
***In vivo* tumor inhibition mechanisms of BPs. (A)** H&E staining images of the liver tumor sections, scale bar is100 µm; **(B)** TUNEL assays at 49 days post-injection for the PBS-, DOX-, and BPs-treated group (scale bar = 100 µm); **(C)** Western blot and semi-quantitative analysis by Image J for apoptosis-related proteins (caspase-3); **(D)** Autophagy-related proteins (LC3-I, LC3-II and p62) extracted from the tumor tissue after the treatment (n = 3). *p < 0.05, significantly different from PBS-treated group.

Histological examination is performed to evaluate the *in vivo* tumor inhibition mechanisms of BPs. The tumor sections from mice sacrificed 49 days after administration are treated with hematoxylin and eosin (H&E) staining and terminal deoxynucleotidyl transferase (TdT)-mediated dUTP-biotin nick end- labeling (TUNEL) assay. As shown in Figure 6A, in comparison with the PBS- and DOX-treated groups, the tumor tissue is loose and shows less nuclear staining in the BPs-treated group suggesting that BPs induce extensive intratumoral necrosis. The TUNEL assay in Figure 6B reveals almost no apoptosis in the PBS- and DOX-treated groups but a large amount of apoptosis occurs as a result of *in situ* BPs inhibition. Apoptosis and necrosis of the tumor tissue induced by BPs are mainly responsible for the tumor suppression effect.

The expression of proteins in the tumor tissue after BPs administration is also investigated. As revealed by Western bolt, the expression of the apoptosis-related protein, cleaved caspase-3, is significantly up-regulated in BPs-treated group (Figure 6C). Apoptosis and autophagy are closely related in terms of the cell fate and impact the curative effects of cancer therapy 66-68, and thus whether BPs can induce autophagy in the tumor tissue is assessed. Figure 6D shows that the protein level of LC3-II in the BPs-treated group is much higher than that in the PBS-treated group indicating that BPs induce autophagy in tumor tissues. The p62 level of the BPs-treated group is also higher than those in other groups indicating that the autophagic flux is blocked by BPs. Consistent with the *in vitro* experiments (Figure 3), BPs may serve as an autophagic inhibitor to impose therapeutic effects on solid tumors 65. The results show that BPs induce apoptosis and autophagy blockage in the hepatocellular carcinomas tissues and subsequently inhibits tumor growth.

### Biosafety of BPs

**Figure 7:**
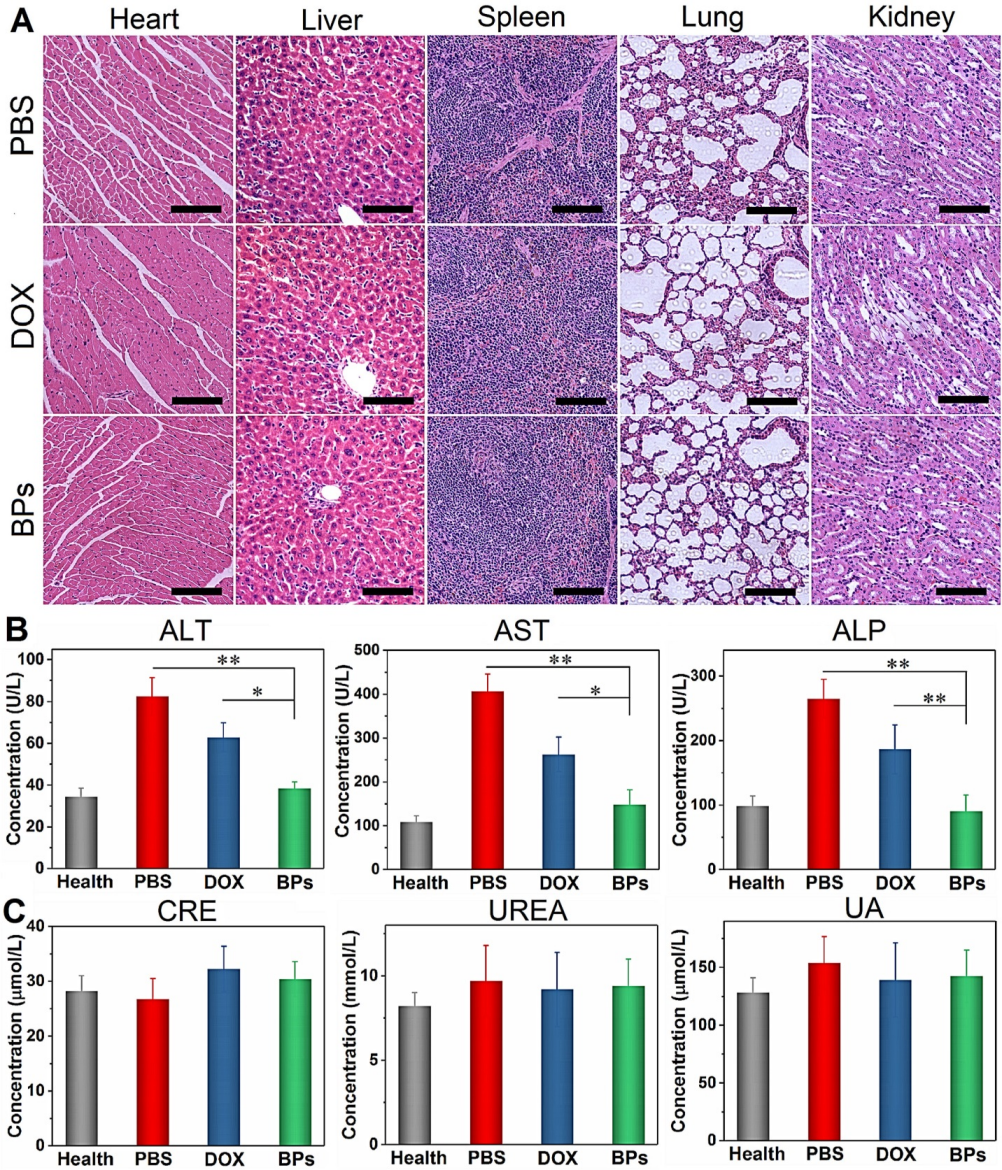
***In vivo* biosafety of BPs. (A)** H&E staining images obtained from the main organs of the PBS-, DOX- and BPs-treated mice after 49 days post-injection (scale bar = 100 µm);** (B)** Blood biochemical analysis of the liver enzymes (ALT, AST and ALP); **(C)** Renal functions (CRE, UREA and UA) of the mice in different groups after 49 days post-injection. The data are shown as means ± s.d.; *p < 0.05, **p < 0.01, significantly different from the PBS- and DOX-treated groups.

The biosafety of BPs as intravenous chemotherapeutic agents is evaluated. At the end of the treatment, no body weight loss is observed from the BPs-treated mice (Figure 5I). H&E staining of the main organs (heart, normal liver, spleen, lung and kidney) suggests that BPs administration does not produce noticeable organ damage or inflammatory lesion (Figure 7A). Additionally, blood biochemical parameters including liver function (alanine transaminase, ALT, aspartate transaminase, AST, and alkaline phosphatase, ALP) and renal function (creatinine, CREA, urea, UREA, and uric acid, UA) analyses indicate no evident hepatic and renal dysfunction. The liver function-related indexes in the BPs-treated group are similar to those of healthy mice on day 49. In contrast, the PBS- or DOX-treated groups show significant increases in these indexes with time (Figure 7B), suggesting impaired liver functions. Moreover, the levels of kidney function- related indexes show no statistical difference among the groups (Figure 7C). The comprehensive studies of the body weight, histological analysis, and serum biomarkers suggest that the BPs treatment imposes no obvious side effects.

### Therapeutic effects of BPs on acute myeloid leukemia

**Figure 8:**
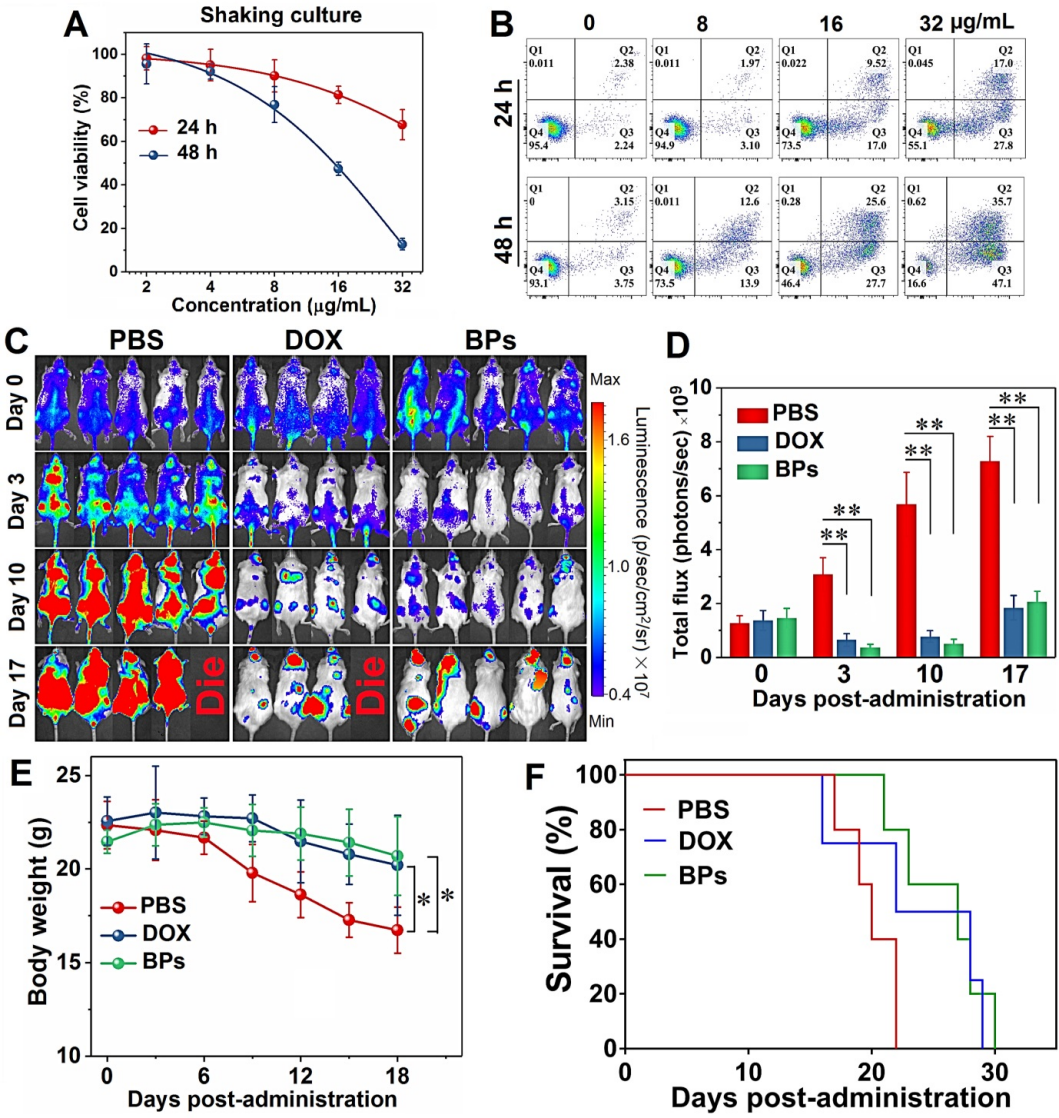
** Chemotherapeutic effects of BPs on acute myeloid leukemia. (A)** Relative cell viability and** (B)** apoptosis assay by Annexin V/PI staining of HL-60 cells as a function of BPs concentration after treatment for 24 or 48 h (n = 6); **(C)** *In vivo* bioluminescence images of the NOD/SCID mice with acute myeloid leukemia taken at different time points after tail vein injection of PBS (control), DOX or BPs. Each of the control and BPs-treated group consists of 5 mice, 4 mice in the DOX-treated group; **(D)** Quantified bioluminescent intensity of mice measured by ROI using the Living Image software; **(E)** Body weight change curves; **(F)** Survival curves of mice. *p < 0.05, **p < 0.01, significantly different from the control group.

In addition to solid tumors, the therapeutic effect of BPs on hematological tumors (typically leukemia) is also preliminarily investigated. The effect of BPs on the cell viability of human HL-60 promyelocytic leukemia cell line is evaluated. Since HL-60 is a type of suspension cultured cell, the culture medium containing BPs is stirred during incubation to facilitate uptake BPs. The CCK-8 assay indicates that about 90% of HL-60 cells are killed after treatment with BPs (32 µg/mL) for 48 h (Figure 8A). The optical microscope photos show that the cells are obviously shrunk and damaged after BPs treatment (Figure S10). Flow cytometry analysis using Annexin V/PI staining confirms that the apoptotic rate of HL-60 cells is 44.8% after 24 h-incubation with BPs and increases to 82.8% after incubation for 48 h (Figure 8B).

The *in vivo* anti-leukemia therapeutic efficacy of BPs is evaluated in the acute myeloid leukemia bearing NOD/SCID mice. As a first-line drug for acute myeloid leukemia, DOX is used to compare the therapeutic effects on acute myeloid leukemia with BPs. The MTD of the NOD/SCID mice is calculated to be 5 mg/kg and 2.5 mg/kg for DOX and BPs, respectively (Figure S9). As shown in Figure 8C, bioluminescent signals are detected from the entire mice in the control group 10 days after intravenous injection. On the other hand, only small signals appear from the BPs-treated mice and the signals are concentrated in certain parts of the body (Figure 8C). The signals intensity of the BPs-treated groups is about 3 times lower than that of the control group at the end of the treatment (Figure 8D). The body weight of the control group begins to decrease significantly after day 9, while that of the BPs-treated group shows almost no change during the treatment period (Figure 8E). The survival curves in Figure 8F show that BPs treatment extends the average survival time of leukemia mice by 29%, which is much shorter than that of the mice bearing the orthotopic liver tumor. The reason is that leukemia cells flourish in the central nervous system such as spinal cord and brain at the later stage of treatment (Figure 8C) eventually causing fatal complication. Comparison of the bioluminescence signal change, body weight change, and survival time between the mice treated by DOX and BPs (Figure 8) shows that the anti-leukemia efficacy of BPs is at least no worse than that of DOX. In fact, the therapy with BPs for leukemia can be improved if BPs can be delivered to the central nervous system hideouts by appropriate modification 69. All in all, these results clearly indicate that BPs exhibit promising chemotherapeutic efficacy not only in solid tumors but also in the hematological tumors.

## Conclusions

In this study, the chemotherapeutic effects of BPs in cancer therapy are assessed systematically. The* in vitro* studies confirm that BPs produce killing effects in cancerous HepG2 cells but are biocompatible in normal QSG-7701 cells due to the high intracellular uptake by cancer cells. Subsequent *in vivo* biodistribution assays show that BPs have tissue-specific affinity in organs such as the liver, lung and spleen and passively accumulate in the orthotopic liver tumor site. *In vivo* anti-tumor experiments demonstrate the better efficacy of BPs compared to the traditional chemotherapeutic agent, DOX, in suppressing tumor growth by apoptosis- and autophagy-mediated cell death of tumors. As a result, the average survival time of BPs-treated mice is prolonged by at least two months. Inspired by excellent therapeutic effect of solid tumor as well as good biosafety determined by H&E staining and blood analysis, the treatment of hematological tumor by BPs is investigated and our preliminary results show that BPs inhibit tumor growth of acute myeloid leukemia *in vivo* as well. Our study demonstrates the great potential of BPs as bioactive nanomaterials for *in vivo* cancer chemotherapy and provides insights into the development of inorganic nanodrugs for cancer therapy.

## Supplementary Material

Supplementary figures.

## Abbreviations

ALP: alkaline phosphatase
ALT: alanine transaminase
AST: aspartate transaminase
BPs: black phosphorus nanosheets
CCK-8: Cell Counting Kit-8
CREA: creatinine
DLS: dynamic light scattering
DOX: doxorubicin
EPR: permeability and retention
HPLC: high performance liquid chromatography
MTD: maximum tolerated dose
RES: reticuloendothelial system
ROI: region of interest
TEM: transmission electron microscopy
UA: uric acid

## References

[1] Lopez-Gomez M, Malmierca E, de Gorgolas M, Casado E (2013). Cancer in developing countries: The next most preventable pandemic. The global problem of cancer. Crit Rev Oncol Hematol.

[2] Bray F, Jemal A, Grey N, Ferlay J, Forman D (2012). Global cancer transitions according to the Human Development Index (2008-2030): a population-based study. Lancet Oncol.

[3] Balogh J, Victor D, Asham EH, Burroughs SG, Boktour M, Saharia A (2016). Hepatocellular carcinoma: a review. J Hepatocell Carcinoma.

[4] Nelson ME, Lahiri S, Chow JD, Byrne FL, Hargett SR, Breen DS (2017). Inhibition of hepatic lipogenesis enhances liver tumorigenesis by increasing antioxidant defence and promoting cell survival. Nat Commun.

[5] Fa BT, Luo CW, Tang Z, Yan YT, Zhang Y, Yu ZS (2019). Pathway-based biomarker identification with crosstalk analysis for robust prognosis prediction in hepatocellular carcinoma. EBioMedicine.

[6] Huo YR, Eslick GD (2015). Transcatheter Arterial Chemoembolization Plus Radiotherapy Compared With Chemoembolization Alone for Hepatocellular Carcinoma: A Systematic Review and Meta-analysisTranscatheter Arterial Chemoembolization Plus RadiotherapyTranscatheter Arterial Chemoembolization Plus Radiotherapy. JAMA Oncol.

[7] Yamauchi K, Yang M, Hayashi K, Jiang P, Yamamoto N, Tsuchiya H (2008). Induction of Cancer Metastasis by Cyclophosphamide Pretreatment of Host Mice: An Opposite Effect of Chemotherapy. Cancer Res.

[8] Oikawa T, Sawara K, Kuroda H, Takikawa Y (2017). Sorafenib treatment for advanced hepatocellular carcinoma: Effectiveness, safety, and controversial points. J Clin Oncol.

[9] Li JN, Xu WG, Li D, Liu TJ, Zhang YS, Ding JX (2018). Locally Deployable Nanofiber Patch for Sequential Drug Delivery in Treatment of Primary and Advanced Orthotopic Hepatomas. ACS Nano.

[10] Moorthi C, Manavalan R, Kathiresan K (2011). Nanotherapeutics to Overcome Conventional Cancer Chemotherapy Limitations. J Pharm Pharm Sci.

[11] Chen YJ, Wu YK, Sun BB, Liu SJ, Liu HY (2017). Two-Dimensional Nanomaterials for Cancer Nanotheranostics. Small.

[12] Lucky SS, Soo KC, Zhang Y (2015). Nanoparticles in Photodynamic Therapy. Chem Rev.

[13] Johnstone TC, Suntharalingam K, Lippard SJ (2016). The Next Generation of Platinum Drugs: Targeted Pt(II) Agents, Nanoparticle Delivery, and Pt(IV) Prodrugs. Chem Rev.

[14] Meng XD, Liu ZQ, Cao Y, Dai WH, Zhang K, Dong HF (2017). Fabricating Aptamer-Conjugated PEGylated-MoS2/Cu1.8S Theranostic Nanoplatform for Multiplexed Imaging Diagnosis and Chemo-Photothermal Therapy of Cancer. Adv Funct Mater.

[15] Shao JD, Xie HH, Huang H, Li ZB, Sun ZB, Xu YH (2016). Biodegradable black phosphorus-based nanospheres for in vivo photothermal cancer therapy. Nat Commun.

[16] Russier J, Leon V, Orecchioni M, Hirata E, Virdis P, Fozza C (2017). Few-Layer Graphene Kills Selectively Tumor Cells from Myelomonocytic Leukemia Patients. Angew Chem Int Ed Engl.

[17] Liu C, Hu XJ, Li XJ, Zhou YF, Wang H, Fan CH (2019). Reprogramming of cancer invasiveness and macrophage education via a nanostructured antagonist of the TGFβ receptor. Mater Horiz.

[18] Sun ZB, Xie HH, Tang SY, Yu XF, Guo ZN, Shao JD (2015). Ultrasmall Black Phosphorus Quantum Dots: Synthesis and Use as Photothermal Agents. Angew Chem Int Ed Engl.

[19] Wang H, Yang XZ, Shao W, Chen SC, Xie JF, Zhang XD (2015). Ultrathin Black Phosphorus Nanosheets for Efficient Singlet Oxygen Generation. J Am Chem Soc.

[20] Chen WS, Ouyang J, Liu H, Chen M, Zeng K, Sheng JP (2017). Black Phosphorus Nanosheet-Based Drug Delivery System for Synergistic Photodynamic/Photothermal/Chemotherapy of Cancer. Adv Mater.

[21] Zhou WH, Cui HD, Ying LM, Yu XF (2018). Enhanced Cytosolic Delivery and Release of CRISPR/Cas9 by Black Phosphorus Nanosheets for Genome Editing. Angew Chem Int Ed Engl.

[22] Sun CX, Wen L, Zeng JF, Wang Y, Sun Q, Deng LJ (2016). One-pot solventless preparation of PEGylated black phosphorus nanoparticles for photoacoustic imaging and photothermal therapy of cancer. Biomaterials.

[23] Wu F, Zhang M, Chu XH, Zhang QC, Su YT, Sun BH (2019). Black phosphorus nanosheets-based nanocarriers for enhancing chemotherapy drug sensitiveness via depleting mutant p53 and resistant cancer multimodal therapy. Chem Eng J.

[24] Zong SF, Wang LL, Yang ZY, Wang H, Wang ZY, Cui YP (2019). Black Phosphorus-Based Drug Nanocarrier for Targeted and Synergetic Chemophotothermal Therapy of Acute Lymphoblastic Leukemia. ACS Appl Mater Interfaces.

[25] Ru CJ, Wey YK, Yu CJ, Azadeh N, Yang L, Jie X (2018). Black Phosphorus and its Biomedical Applications. Theranostics.

[26] Zhou Y, Wang DP, Zhang YM, Chitgupi U, Geng JM, Wang YH (2016). A Phosphorus Phthalocyanine Formulation with Intense Absorbance at 1000 nm for Deep Optical Imaging. Theranostics.

[27] Mei LQ, Zhu S, Yin WY, Chen CY, Nie GJ, Gu ZJ (2020). Two-dimensional nanomaterials beyond graphene for antibacterial applications: current progress and future perspectives. Theranostics.

[28] Ou W, Byeon JH, Soe ZC, Kim BK, Thapa RK, Gupta B (2019). Tailored Black Phosphorus for Erythrocyte Membrane Nanocloaking with Interleukin-1alpha siRNA and Paclitaxel for Targeted, Durable, and Mild Combination Cancer Therapy. Theranostics.

[29] Zhang XJ, Zhang ZM, Zhang SY, Li DY, Ma W, Ma CX (2017). Size Effect on the Cytotoxicity of Layered Black Phosphorus and Underlying Mechanisms. Small.

[30] Mo JB, Xie QY, Wei W, Zhao J (2018). Revealing the immune perturbation of black phosphorus nanomaterials to macrophages by understanding the protein corona. Nat Commun.

[31] Zhou QH, Chen Q, Tong YL, Wang JL (2016). Light-Induced Ambient Degradation of Few-Layer Black Phosphorus: Mechanism and Protection. Angew Chem Int Ed Engl.

[32] Zhang TM, Wan YY, Xie HY, Mu Y, Du PW, Wang D (2018). Degradation Chemistry and Stabilization of Exfoliated Few-Layer Black Phosphorus in Water. J Am Chem Soc.

[33] Kawai M, Guth K, Winnikes K, Haist C, Ruegg JC (1987). The Effect of Inorganic-Phosphate on the Atp Hydrolysis Rate and the Tension Transients in Chemically Skinned Rabbit Psoas Fibers. Pflugers Arch.

[34] Martin C, Schulz R, Rose J, Heusch G (1998). Inorganic phosphate content and free energy change of ATP hydrolysis in regional short-term hibernating myocardium. Cardiovasc Res.

[35] Meleti Z, Shapiro IM, Adams CS (2000). Inorganic phosphate induces apoptosis of osteoblast-like cells in culture. Bone.

[36] Di Marco GS, Hausberg M, Hillebrand U, Rustemeyer P, Wittkowski W, Lang D (2008). Increased inorganic phosphate induces human endothelial cell apoptosis in vitro. Am J Physiol Renal Physiol.

[37] Reuter S, Gupta SC, Chaturvedi MM, Aggarwal BB (2010). Oxidative stress, inflammation, and cancer How are they linked?. Free Radic Biol Med.

[38] Moreno-Sanchez R, Rodriguez-Enriquez S, Marin-Hernandez A, Saavedra E (2007). Energy metabolism in tumor cells. FEBS J.

[39] Zhou WH, Pan T, Cui HD, Zhao Z, Chu PK, Yu XF (2019). Black Phosphorus: Bioactive Nanomaterials with Inherent and Selective Chemotherapeutic Effects. Angew Chem Int Ed Engl.

[40] Deng GL, Zeng S, Shen H (2015). Chemotherapy and target therapy for hepatocellular carcinoma: New advances and challenges. World J Hepatol.

[41] Lin SB, Hoffmann K, Schemmer P (2012). Treatment of Hepatocellular Carcinoma: A Systematic Review. Liver Cancer.

[42] Dombret H, Gardin C (2016). An update of current treatments for adult acute myeloid leukemia. Blood.

[43] Yang CL, Wu TT, Qin YT, Qi Y, Sun Y, Kong M (2018). A facile doxorubicin-dichloroacetate conjugate nanomedicine with high drug loading for safe drug delivery. Int J Nanomedicine.

[44] Guo DD, Shi CY, Wang X, Wang LL, Zhang SL, Luo JT (2017). Riboflavin-containing telodendrimer nanocarriers for efficient doxorubicin delivery: High loading capacity, increased stability, and improved anticancer efficacy. Biomaterials.

[45] Lee CC, Gillies ER, Fox ME, Guillaudeu SJ, Frechet JMJ, Dy EE (2006). A single dose of doxorubicin-functionalized bow-tie dendrimer cures mice bearing C-26 colon carcinomas. Proc Natl Acad Sci U S A.

[46] Geng SY, Yang B, Wang GW, Qin G, Wada S, Wang JY (2014). Two cholesterol derivative-based PEGylated liposomes as drug delivery system, study on pharmacokinetics and drug delivery to retina. Nanotechnology.

[47] Guo ZN, Zhang H, Lu SB, Wang ZT, Tang SY, Shao JD (2015). From Black Phosphorus to Phosphorene: Basic Solvent Exfoliation, Evolution of Raman Scattering, and Applications to Ultrafast Photonics. Adv Funct Mater.

[48] Wood JD, Wells SA, Jariwala D, Chen K-S, Cho E, Sangwan VK (2014). Effective Passivation of Exfoliated Black Phosphorus Transistors against Ambient Degradation. Nano Lett.

[49] Edmonds MT, Tadich A, Carvalho A, Ziletti A, O'Donnell KM, Koenig SP (2015). Creating a Stable Oxide at the Surface of Black Phosphorus. ACS Appl Mater Interfaces.

[50] Artel V, Guo Q, Cohen H, Gasper R, Ramasubramaniam A, Xia F (2017). Protective molecular passivation of black phosphorus. NPJ 2D Mater Appl.

[51] Li J, Chen YC, Tseng YC, Mozumdar S, Huang L (2010). Biodegradable calcium phosphate nanoparticle with lipid coating for systemic siRNA delivery. J Control Release.

[52] Tao W, Zhu XB, Yu XH, Zeng XW, Xiao QL, Zhang XD (2017). Black Phosphorus Nanosheets as a Robust Delivery Platform for Cancer Theranostics. Adv Mater.

[53] Levine B (2007). Cell biology - Autophagy and cancer. Nature.

[54] Kondo Y, Kanzawa T, Sawaya R, Kondo S (2005). The role of autophagy in cancer development and response to therapy. Nat Rev Cancer.

[55] Zhou CH, Zhong W, Zhou J, Sheng FG, Fang ZY, Wei Y (2012). Monitoring autophagic flux by an improved tandem fluorescent-tagged LC3 (mTagRFP-mWasabi-LC3) reveals that high-dose rapamycin impairs autophagic flux in cancer cells. Autophagy.

[56] Lopez G, Torres K, Lev D (2011). Autophagy blockade enhances HDAC inhibitors' pro-apoptotic effects Potential implications for the treatment of a therapeutic-resistant malignancy. Autophagy.

[57] Ng S, Wu YT, Chen B, Zhou J, Shen HM (2011). Impaired autophagy due to constitutive mTOR activation sensitizes TSC2-null cells to cell death under stress. Autophagy.

[58] Mancias JD, Kimmelman AC (2011). Targeting Autophagy Addiction in Cancer. Oncotarget.

[59] Zhang L, Qiang PF, Yu JT, Miao YM, Chen ZQ, Qu J (2019). Identification of compound CA-5f as a novel late-stage autophagy inhibitor with potent anti-tumor effect against non-small cell lung cancer. Autophagy.

[60] Kollmar O, Schilling MK, Menger MD (2004). Experimental liver metastasis: Standards for local cell implantation to study isolated tumor growth in mice. Clin Exp Metastas.

[61] Bourquin J, Milosevic A, Hauser D, Lehner R, Blank F, Petri-Fink A (2018). Biodistribution, Clearance, and Long-Term Fate of Clinically Relevant Nanomaterials. Adv Mater.

[62] Sa LTM, Albernaz MdS, Patricio BFdC, Junior MVF, Coelho BF, Bordim A (2012). Biodistribution of nanoparticles: Initial considerations. J Pharm Biomed Anal.

[63] Fabian E, Landsiedel R, Ma-Hock L, Wiench K, Wohlleben W, van Ravenzwaay B (2008). Tissue distribution and toxicity of intravenously administered titanium dioxide nanoparticles in rats. Archf Toxicol.

[64] He L, Tian DA, Li PY, He XX (2015). Mouse models of liver cancer: Progress and recommendations. Oncotarget.

[65] Cui ZF, Zhang Y, Xia K, Yan QL, Kong HT, Zhang JC (2018). Nanodiamond autophagy inhibitor allosterically improves the arsenical-based therapy of solid tumors. Nat Commun.

[66] Levine B, Abrams J (2008). p53: The Janus of autophagy?. Nat Cell Biol.

[67] Maiuri MC, Criollo A, Kroemer G (2010). Crosstalk between apoptosis and autophagy within the Beclin 1 interactome. EMBO J.

[68] Luo S, Rubinsztein DC (2007). Atg5 and Bcl-2 provide novel insights into the interplay between apoptosis and autophagy. Cell Death Differ.

[69] Soni G, Yadav KS (2015). Applications of nanoparticles in treatment and diagnosis of leukemia. Mater Sci Eng C Mater Biol Appl.

