# Supplementary material for: Bioactive phospho-therapy with black phosphorus for in vivo tumor suppression
Source: Theranostics. 2020 Mar 26;10(11):4720–36. doi: 10.7150/thno.43092 (PMC7163432; doi:10.7150/thno.43092)
Supplement: Supplementary file 1 — Supplementary figures. [file thnov10p4720s1.pdf]

Supporting Information

Bioactive phospho-therapy with black phosphorus for *in vivo* tumor suppression

Shengyong Geng [1,2], Ting Pan[1], Wenhua Zhou[1], Haodong Cui[1], Lie Wu[1], Zhibin Li[1], Paul K. Chu[3], and Xue-Feng Yu[1,4]*

[1] Materials and Interfaces Center, Shenzhen Institutes of Advanced Technology, Chinese Academy of Sciences, Shenzhen 518055, China

[2] Medical Oncology, Shenzhen People's Hospital, the Second Clinical Medical College of Jinan University, Shenzhen 518055, China

[3] Department of Physics, Department of Materials Science and Engineering, and Department of Biomedical Engineering, City University of Hong Kong, Tat Chee Avenue, Kowloon, Hong Kong, China

[4] Institute of Environment and Health, Jianghan University, Wuhan 430056, China

* Corresponding author: Xue-Feng Yu

Tel: +86-13971313505

Email: xf.yu@siat.ac.cn

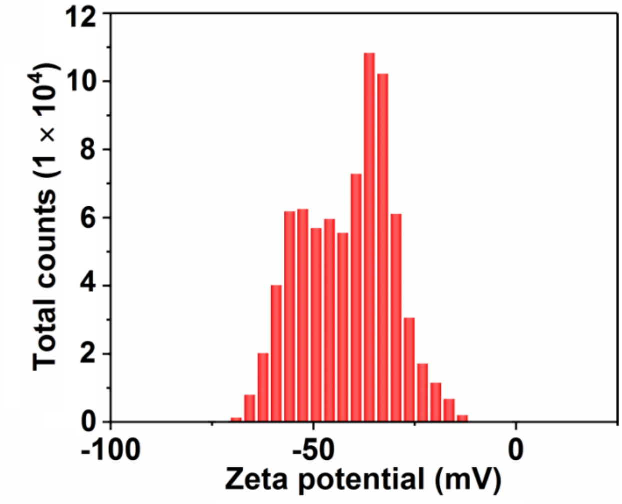

Figure S1. Zeta potentials of BPs.

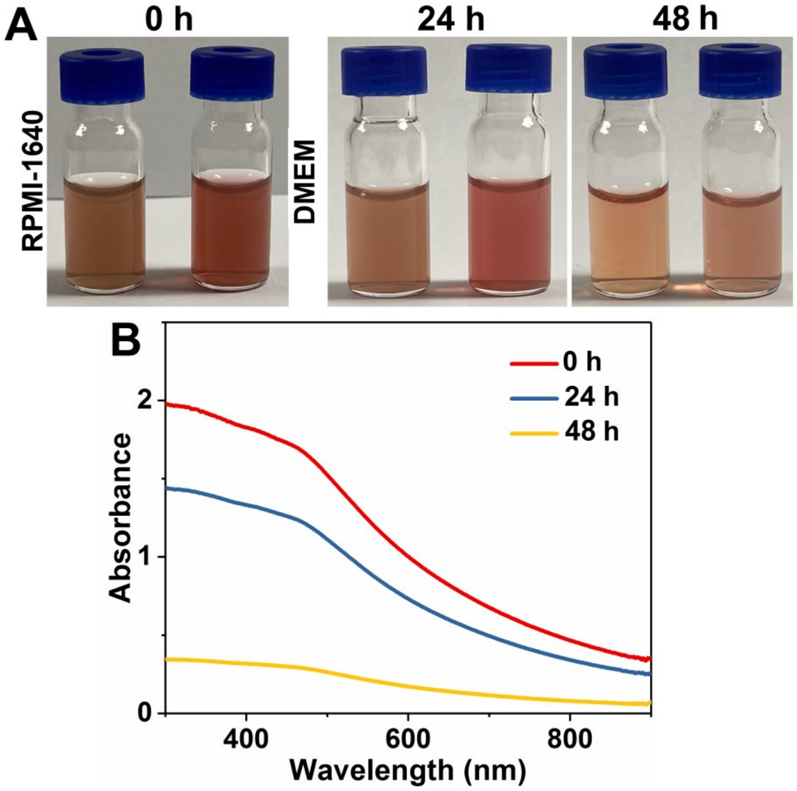

Figure S2. Stability evaluation of BPs: (A) Photographs and (B) Absorption spectra of the cell culture medium (RPMI-1640 and DMEM) containing 10% serum and 20 μg/mL of BPs.

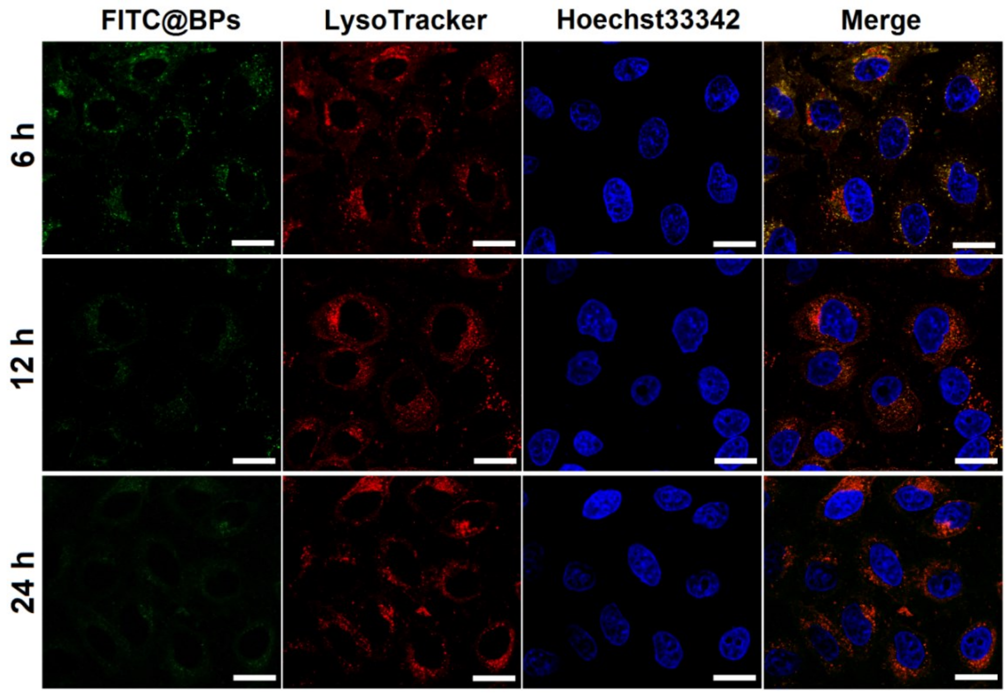

Figure S3. Confocal fluorescence images of QSG-7701 cells treated with BPs (4 μg/mL) for different time intervals. The green and red fluorescence images show FITC and LysoTracker Red DND-99, respectively. The scale bar is 20 μm.

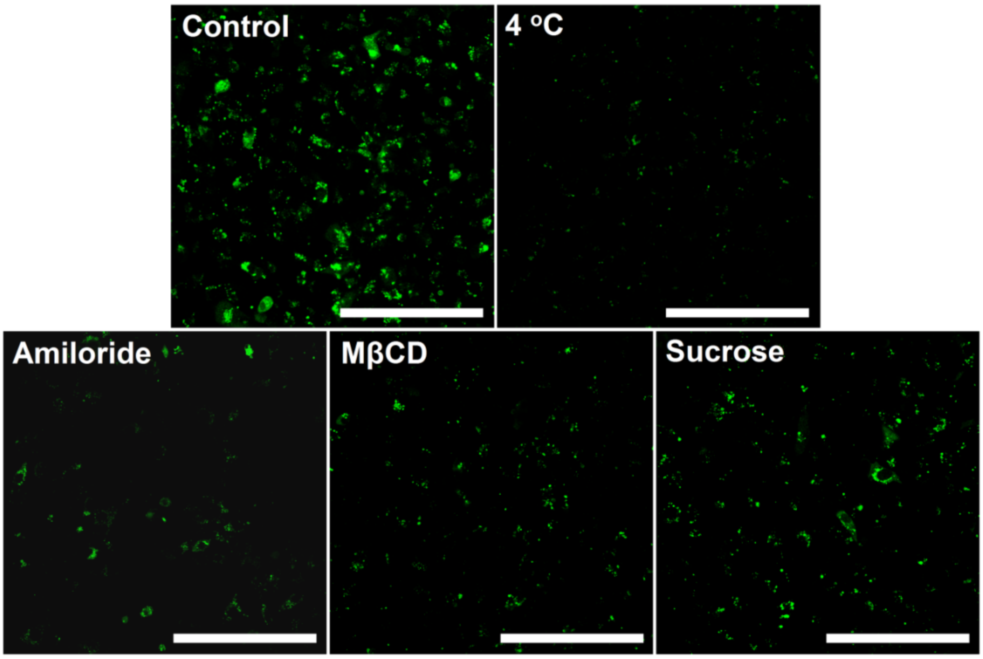

Figure S4. Confocal fluorescence images of HepG2 cells after treatment at low temperature (4 °C) with different endocytosis inhibitors. The scale bar is 100 μm.

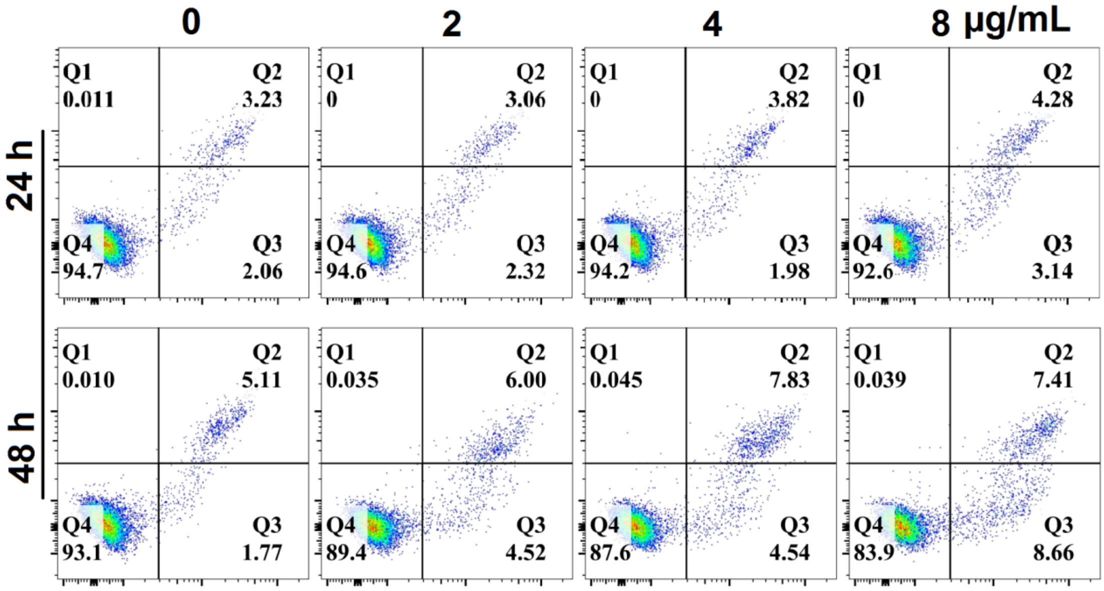

Figure S5. Apoptosis assay of QSG-7701 cells by Annexin V/PI staining after treatment with different concentrations of BPs for 24 or 48 h.

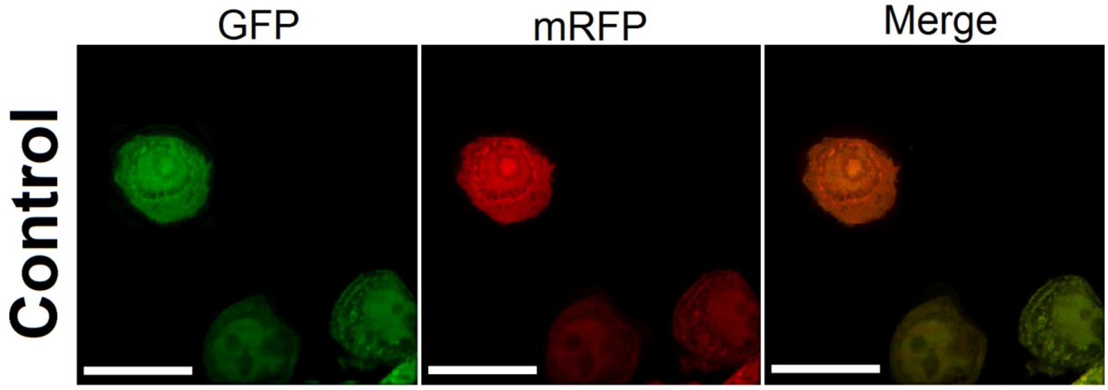

Figure S6. Confocal fluorescent images of mRFP-GFP-LC3-transfected HepG2 cells without treatment with BPs for 48 h (Scale bar = 20 μm).

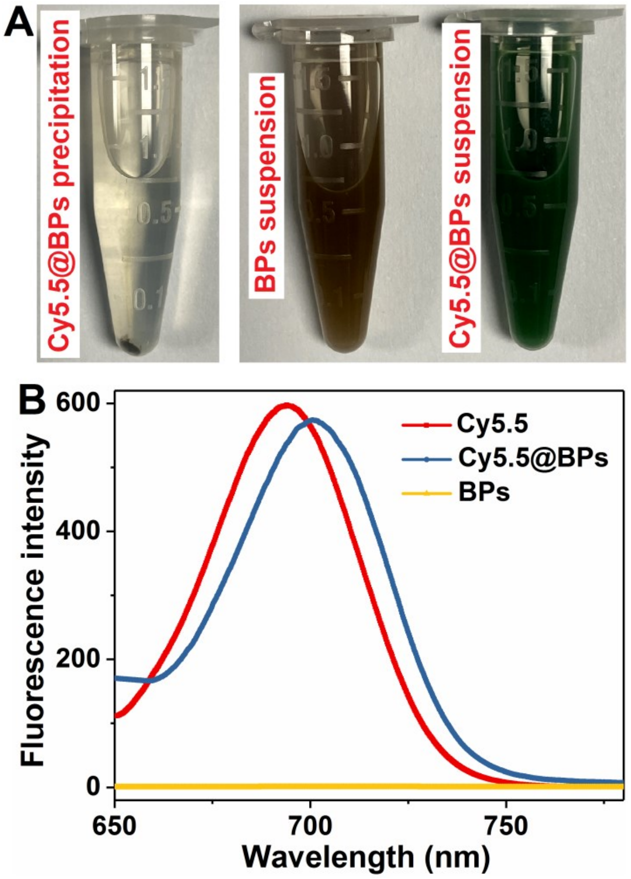

Figure S7. Characterization of Cy5.5-labeled BPs: (A) Photographs of the BPs and Cy5.5-labeled BPs; (B) Fluorescence spectra of Cy5.5 and Cy5.5-labeled BPs (Excitation wavelength = 675 nm). No fluorescence can be observed from the BPs without Cy5.5 labeling.

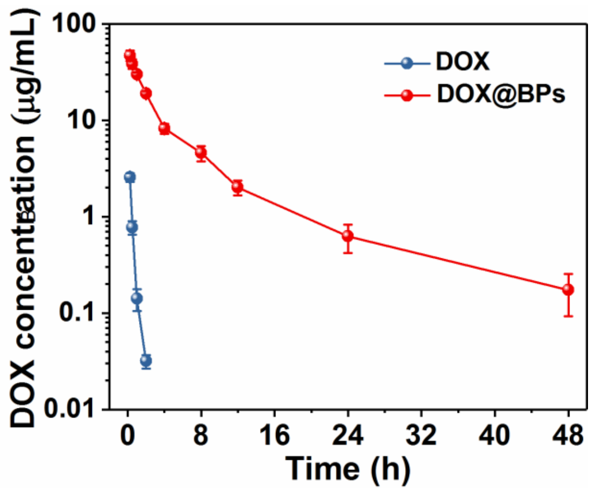

Figure S8. Plasma concentration of DOX after a single intravenous injection of free DOX and DOX-labeled BPs (5 mg/kg, n = 3).

Table S1. Pharmacokinetic parameters of free DOX and DOX-labeled BPs (n = 3).

Parameters	$AUC_{0\text{-}t}$ (h·μg/mL)	C_{max} (μg/mL)	Lz (1/h)	$MRT_{0\text{-}t}$ (h)	CL (mL/h)
Free DOX	0.64 ± 0.1	2.57 ± 0.54	1.82 ± 0.23	0.60 ± 0.08	1856.6 ± 89.5
DOX@BPs	204.6 ± 15.4	47.11 ± 5.63	0.18 ± 0.04	4.36 ± 0.49	5.34 ± 0.97

$AUC_{0\text{-}t}$, total area under the blood concentration versus time curve; C_{max}, maximum plasma concentration; Lz, elimination rate constant; $MRT_{0\text{-}t,}$ mean residence time; CL, total body clearance.

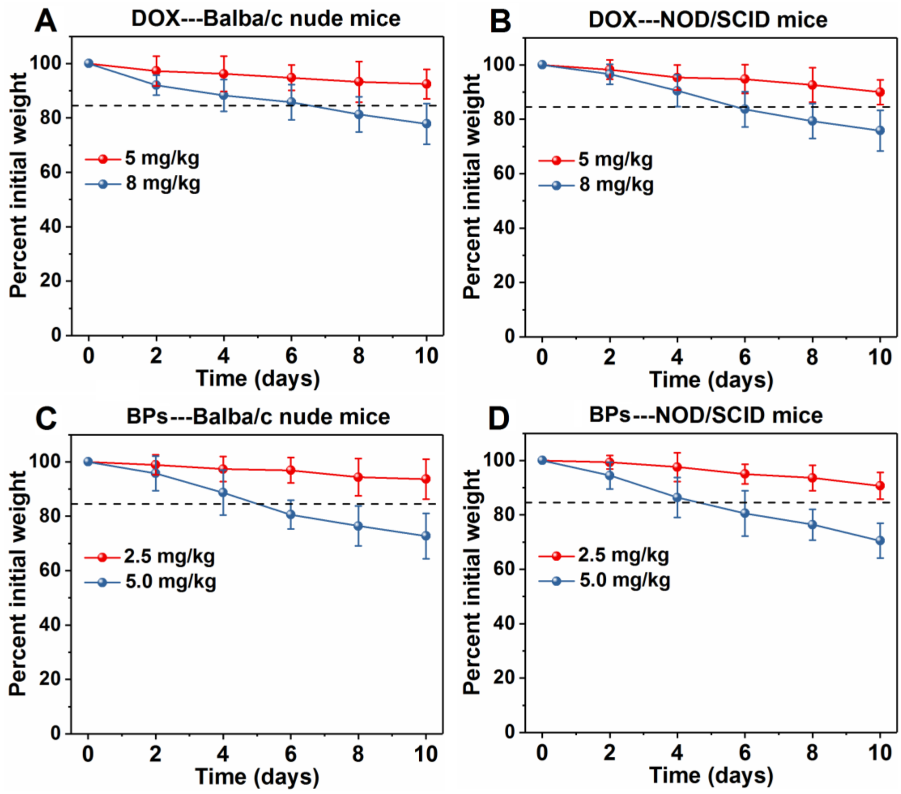

Figure S9. Average mouse weights versus time. The data are presented as the percentage of the initial weights for Balb/c nude mice and NOD/SCID mice treated with DOX (A, B) or BPs (C, D) and n = 3 for each treatment group.

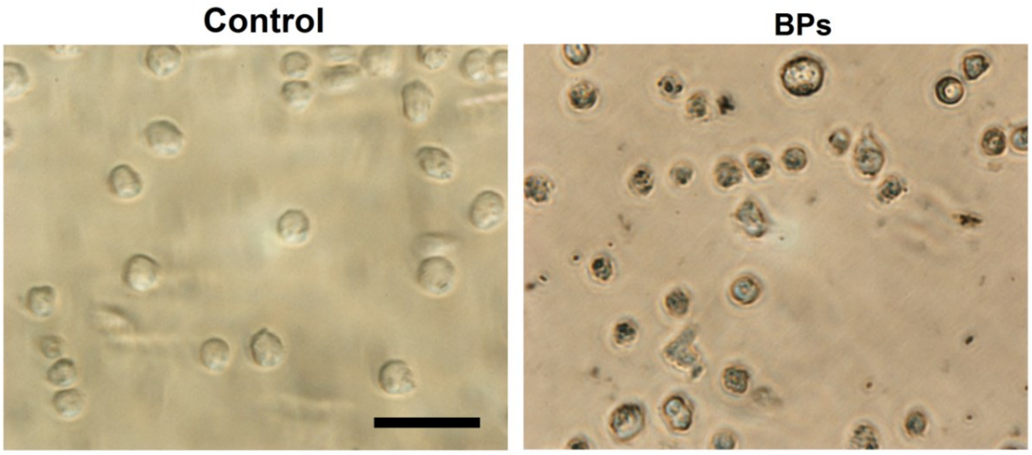

Figure S10. Optical microscopy images of HL-60 cells without/with incubation with BPs (32 μg/mL) for 24 h (Scale bar = 50 μm).
